# Soluble factors released by peripheral blood-derived CAR-NK cells cause bystander myeloid cell activation

**DOI:** 10.3389/fimmu.2024.1519415

**Published:** 2024-12-24

**Authors:** Supreet Khanal, Alan Baer, Md Kamal Hossain, Winston Colon-Moran, Santosh Panthi, Nirjal Bhattarai

**Affiliations:** Tumor Vaccine and Biotechnology Branch, Office of Cellular Therapy and Human Tissues, Office of Therapeutic Products, Center for Biologics Evaluation and Research, United States Food and Drug Administration (U.S. FDA), Silver Spring, MD, United States

**Keywords:** NK cell, CAR (chimeric antigen receptor), inflammation, CRS, neurotoxicity

## Abstract

**Introduction:**

CAR-T cell therapy is associated with life-threatening inflammatory toxicities, partly due to the activation and secretion of inflammatory cytokines by bystander myeloid cells (BMCs). However, due to limited clinical data, it is unclear whether CAR-NK cells cause similar toxicities.

**Methods:**

We characterized the soluble factors (SFs) released by activated human CAR-T and CAR-NK cells and assessed their role in BMC activation (BMCA).

**Results:**

We found that SFs from both activated, peripheral blood-derived CAR-T (PB-CAR-T) and CAR-NK (PB-CAR-NK) cells induced BMCA; however, PB-CAR-NK cells caused significantly lower BMCA compared to PB-CAR-T cells. Interestingly, SFs from cord-blood-derived (CB) NK cells caused little to no BMCA, consistent with previous clinical studies showing minimal inflammatory toxicity with CB-CAR-NK cells. Comparative analysis of SFs released by PB-NK and PB-CAR-NK cells following CAR-dependent and CAR-independent activation revealed several candidate factors with the potential to cause BMCA. Antibody-mediated neutralization studies identified a combination of four factors that contribute to PB-CAR-NK cell-mediated BMCA. siRNA-mediated knockdown studies confirmed that inactivating these four factors in PB-CAR-NK cells significantly reduces BMCA. Importantly, neutralization or knockdown of these four factors did not affect CAR-NK cell potency.

**Discussion:**

These data suggest that specific SFs released by PB-CAR-NK cells activate BMCs and have the potential to contribute to inflammatory toxicities. Furthermore, inactivation of these four factors in PB-CAR-NK cells could reduce inflammatory toxicities and improve safety of PB-CAR-NK cell therapy without compromising potency.

## Introduction

CAR-T cell therapy has revolutionized the treatment of various cancers; however, safety issues, such as the development of inflammatory toxicities (e.g., cytokine release syndrome [CRS] and immune effector cell-associated neurotoxicity syndrome [ICANS]), present a significant safety risk (1, 2). The timing and severity of these toxicities can depend on several factors, such as CAR construct design, disease type, tumor burden, age, and comorbidities (3). CAR-NK cell therapy is another type of cell-based gene therapy emerging in the field of cancer immunotherapy. Due to their intrinsic ability to kill target cells via multiple mechanisms without sensitization or HLA restriction, along with a comparatively better safety profile and the feasibility of allogeneic off-the-shelf treatment, CAR-NK cells are being widely developed as an alternative to CAR-T cells.

To manufacture CAR-NK cells, NK cells are obtained from various sources, such as peripheral blood, cord blood, or by differentiation from hematopoietic stem cells or induced pluripotent stem cells (4–6). Variability in the NK cell source can contribute to differences in CAR-NK cell expansion, maturation, potency, and safety (4–8). For example, significant differences have been observed in the surface expression of adhesion molecules on NK cells derived from cord blood compared to those from peripheral blood (5, 9). NK cells derived from human umbilical cord blood exhibit lower activity due to reduced expression of adhesion molecules compared to NK cells from peripheral blood (5, 10). Given the limited published clinical data on CAR-NK cell therapy, it is unclear whether differences in starting NK cell material impact clinical outcomes. For instance, in a clinical study that used cord blood-derived NK cells to manufacture CAR-NK cells for the treatment of relapsed or refractory CD19+ lymphoid tumors, none of the 11 participants developed inflammatory toxicities such as CRS or ICANS (11). However, in another preclinical study, treatment with cord blood-derived CAR-NK cells resulted in high levels of inflammatory cytokines in the serum, leading to animal death (12). When the NK-92 cell line was used to generate CAR-NK cells for the treatment of advanced non-small cell lung cancer, CRS was observed in one of the two patients (13). Several other clinical studies have also documented the development of inflammatory toxicities following NK cell therapy (14, 15). Given the variability in the development of inflammatory toxicities during NK cell therapy, it is important to understand the factors that contribute to such toxicities during CAR-NK cell therapy.

During CAR-T cell therapy, the activation of bystander myeloid cells (e.g., monocytes, macrophages) and the production of inflammatory cytokines, such as IL-6 and IL-1β, by these activated myeloid cells is an important early step in the development of inflammatory toxicities (13, 16, 17). Currently, it is unclear whether CAR-NK cells have the potential to cause similar inflammatory toxicities. Upon activation, NK cells also secrete a variety of soluble factors that can activate bystander myeloid cells and have the potential to cause inflammatory toxicities (4, 8). Identifying these key inflammatory factors secreted by NK cells can provide novel insights into the mechanisms driving inflammatory toxicities during CAR-NK cell therapy. It may also lead to the development of better strategies to treat inflammatory toxicities or improve the safety of CAR-NK cells by inhibiting these inflammatory factors during their manufacture.

In this study, we characterized the soluble factors released by activated CAR-NK cells obtained from different sources and investigated their potential to induce bystander myeloid cell activation. We identified key inflammatory factors secreted by activated peripheral blood-derived (PB)-CAR-NK cells that induce myeloid cell activation, which may contribute to inflammatory toxicities. Furthermore, we demonstrated that antibody-mediated neutralization or siRNA-mediated knockdown of these key inflammatory factors significantly reduces PB-CAR-NK cell-mediated myeloid cell activation without affecting their potency.

## Results

### Peripheral blood-derived CAR-T cells are more inflammatory than peripheral blood-derived CAR-NK cells

CAR-T cell therapy is associated with severe, life-threatening inflammatory and systemic toxicities. Limited clinical data suggest that CAR-NK cell therapy may be safer than CAR-T cell therapy (11, 18); however, factors such as cellular origin, patient characteristics, disease indication, antigen burden, and cellular dose may also affect safety outcomes in both CAR-T and CAR-NK cell therapies. Previous studies have found that soluble factors released by CAR-T cells activate bystander myeloid cells (1, 16, 19). Furthermore, the activation of bystander myeloid cells and the release of inflammatory cytokines, such as IL-6 and IL-1β, by these activated myeloid cells are important early steps in the manifestation of inflammatory toxicities (1, 19). Unlike CAR-T cells, it is unknown whether the soluble factors released by CAR-NK cells cause bystander myeloid cell activation and contribute to inflammatory toxicities.

**Figure 1:**
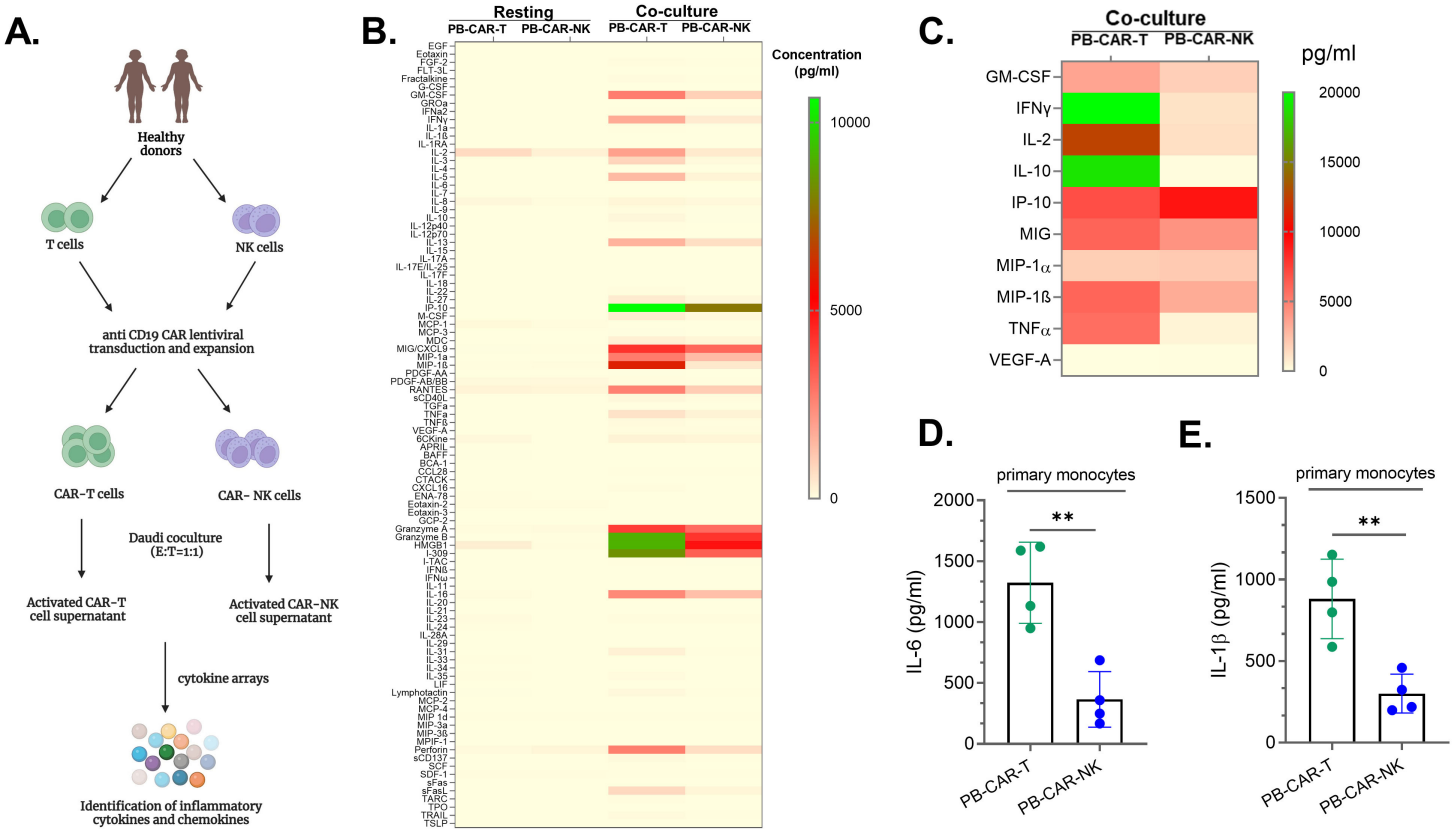
Peripheral blood (PB)-derived CAR-T cells are more inflammatory than peripheral blood (PB)-derived CAR-NK cells. **(A)** Schematic of experimental steps followed for analysis of the supernatants obtained from PB-CAR-NK and PB-CAR-T cells for inflammatory cytokines/chemokines using cytokine arrays. **(B)** Heatmap of 96 inflammatory cytokines/chemokines present in the supernatant of resting or Daudi co-culture activated anti-CD19 PB-CAR-NK cells and anti-CD19 PB-CAR-T cells (E: T= 1:1). Cytokine/chemokine levels (expressed in pg/ml) are represented by a color gradient, ranging from yellow (no expression) to green (highest concentration). **(C)** Heat map of 10 CRS-related inflammatory cytokines present in the supernatant of activated PB-NK cells and PB-CAR-T cells. **(D)** IL-6 and **(E)** IL-1β secreted by primary monocytes following treatment with activated PB-CAR-NK or PB-CAR-T cell supernatant. **P<0.01. Each dot represents one donor.

To characterize the soluble factors released by both activated CAR-T and CAR-NK cells and assess their ability to cause myeloid cell activation, anti-CD19 CAR-T and anti-CD19 CAR-NK cells were manufactured using peripheral blood (PB)-derived human T and NK cells (**Figure 1A**). Anti-CD19 CAR-expressing cells were activated following co-culture with CD19+ Daudi cells (E: T= 1:1). After 24 hours of activation, cell-free supernatants from resting, unstimulated, or co-culture activated PB-CAR-T and PB-CAR-NK cells were analyzed for cytokines and chemokines using a multiplex cytokine array (**Figure 1A**). We found that the supernatant from resting PB-CAR-T and PB-CAR-NK cells had very few inflammatory cytokines and chemokines, expressed at very low levels (**Figure 1B**). However, following co-culture activation, PB-CAR-T cells released significantly more inflammatory cytokines and chemokines at higher levels compared to PB-CAR-NK cells (**Figure 1B**). Cytokines that are commonly associated with inflammatory toxicities in patients receiving CAR-T cell therapy (19–21), were also significantly elevated in the supernatant obtained from activated PB-CAR-T cells compared to PB-CAR-NK cells (**Figure 1C**). To confirm these findings, CAR-T and CAR-NK cells were manufactured from T and NK cells obtained from additional donors and subjected to the cytokine array analysis. Consistently, we found that activated PB-CAR-T cells secreted significantly more inflammatory cytokines and chemokines at higher levels than PB-CAR-NK cells (**Supplementary Figure S1A**). Together, these data suggest that PB-CAR-T cells secrete significantly higher levels of inflammatory cytokines compared to PB-CAR-NK cells after activation.

To confirm that the observed differences between PB-CAR-T and PB-CAR-NK cells were not due to differences in CAR-mediated signaling in these cells or any other donor-related factors, T and NK cells were isolated from the same donor’s peripheral blood (PB). Both PB-T and PB-NK cells were activated in a CAR-independent mechanism using PMA and Ionomycin (PMA/Iono), which activate cells through the PKC and calcineurin pathways (22). The expression of two inflammatory cytokines, IFNγ and GM-CSF, was assessed following activation. In each donor studied (N=6), activated PB-T cells secreted significantly higher levels of IFNγ and GM-CSF compared to PB-NK cells from the same donor (**Supplementary Figures S1B, C**). Together, these data suggest that following activation PB-T cells secrete significantly more inflammatory cytokines than PB-NK cells, independent of CAR-mediated signaling.

Next, we investigated whether PB-T cells are more inflammatory than PB-NK cells by assessing their ability to induce bystander myeloid cell activation (BMCA). BMCA was evaluated by measuring the release of IL-6 and IL-1β by myeloid cells (primary human monocytes and the THP-1 monocytic cell line) following incubation with supernatants from resting or activated PB-CAR-T or PB-CAR-NK cells. We found that both IL-6 and IL-1β levels were produced by primary monocytes at significantly higher levels after treatment with equal amount of PB-CAR-T cell supernatant compared to PB-CAR-NK cell supernatant (**Figures 1D, E**). Similarly, when both PB-T and PB-NK cells were obtained from the same donor and activated in a CAR-independent manner (PMA/Iono), supernatants from PB-T cells induced significantly higher levels of BMCA compared to those from PB-NK cells (**Supplementary Figures S1D, E**). We confirmed that IL-6 and IL-1β were secreted by myeloid cells and not by other cell types by activating various other cells used in our studies. None of the non-myeloid cells used in our studies secreted IL-6 and IL-1β following activation (**Supplementary Figure S2**). Furthermore, myeloid cells (THP-1) stimulated with PMA/Iono also did not secrete IL-6 and IL-1β (**Supplementary Figure S2**) suggesting that residual amount of PMA/Iono that may be present in the PMA/Iono activated T and NK cell supernatant does not activate myeloid cells. Only when stimulated with PMA/Iono activated NK cell supernatant, THP-1 cells secreted IL-6 and IL-1β (**Supplementary Figure S2**). Thus, IL-6 and IL-1β released by myeloid cells in these studies are due to activation of these cells by soluble factors present in the activated cell supernatant.

Together, these data suggest that PB-CAR-T cells are more inflammatory and cause significantly higher levels of bystander myeloid cell activation compared to PB-CAR-NK cells.

### Peripheral blood-derived NK cells are more inflammatory than cord blood-derived NK cells

Recent clinical studies have reported little to no inflammatory toxicities (CRS, ICANS) in patients with B cell malignancies following treatment with cord blood (CB)-derived anti-CD19 CAR-NK cells, suggesting that CB-CAR-NK cells may not cause bystander myeloid cell activation (BMCA) (11, 18). Although we observed that peripheral blood (PB)-derived CAR-NK cells secreted lower levels of inflammatory cytokines, leading to reduced BMCA compared to PB-CAR-T cells (**Figure 1**), a considerable amount of inflammatory cytokines and BMCA was still detected with PB-CAR-NK cells. This led us to hypothesize that compared CB-NK cells, PB-NK cells may cause higher levels of BMCA and are more inflammatory.

**Figure 2:**
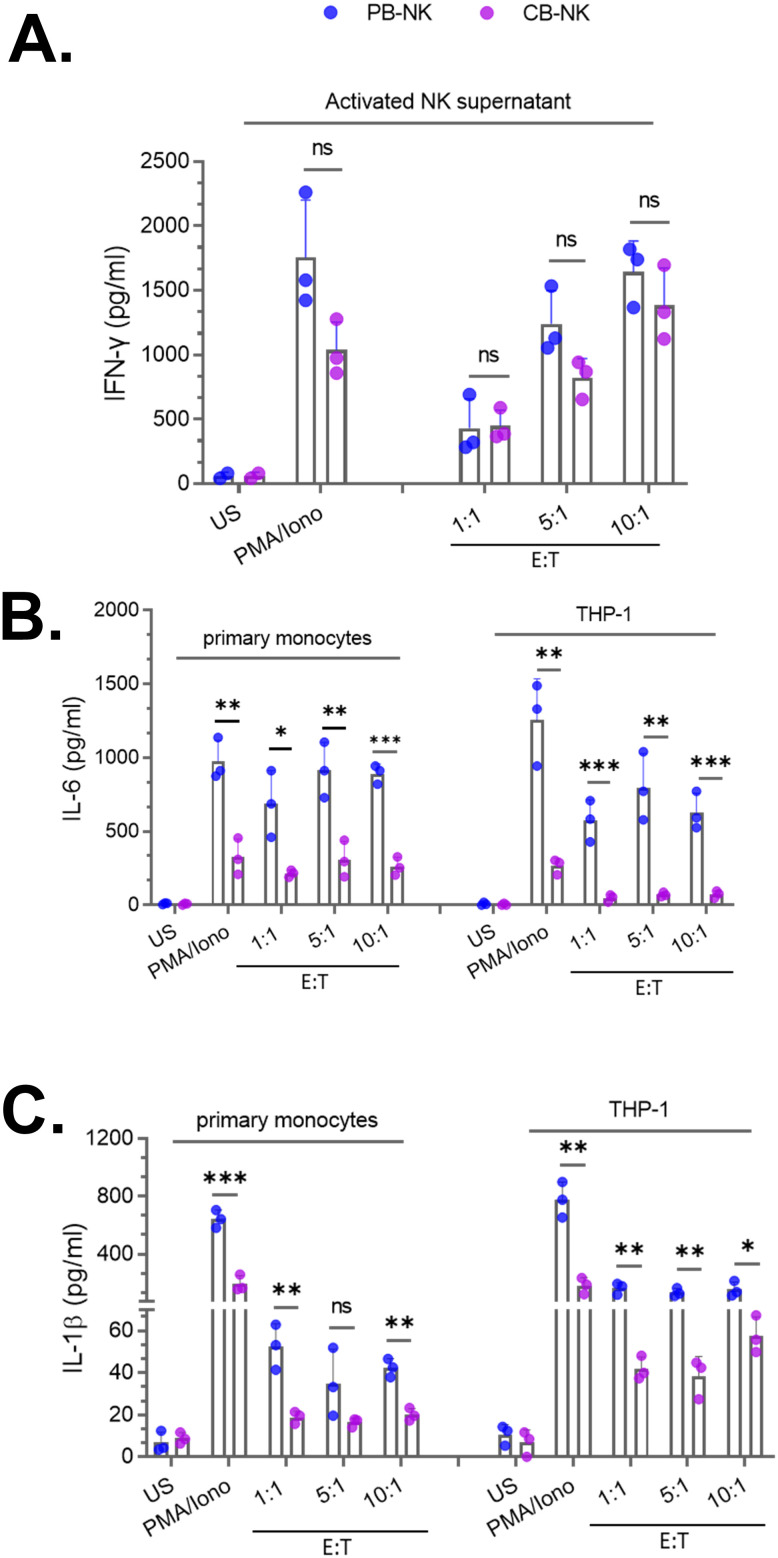
Peripheral blood-derived NK cells (PB-NK) induce significantly higher levels of myeloid cell activation compared to cord blood-derived NK cells (CB-NK) **(A)** Supernatants from unstimulated (US) and activated CB-NK or PB-NK cells (activated using PMA/Ionomycin or after co-culture with Daudi target cells at various effector to target (E: T) cell ratios were assessed for IFNγ. Primary monocytes and THP-1 cells were treated with NK cell supernatants from panel A to assess myeloid activation by measuring IL-6 and IL-1β released by myeloid cells. IL-6 **(B)** and IL-1β **(C)** released by primary monocytes and THP-1 cells were measured by ELISA. *P<0.05, **P<0.01, ***P<0.001. Each dot represents one donor.

To test this hypothesis, CB-NK and PB-NK cells were obtained from healthy donors and activated following co-culture with Daudi target cells at various effector-to-target (E: T) ratios or by PMA and Ionomycin (PMA/Iono). After 24 hours, cellular activation was assessed by measuring IFNγ released in the supernatant. Both CB-NK and PB-NK cells secreted comparable amounts of IFNγ (**Figure 2A**), suggesting that unlike PB-T cells, both CB-NK and PB-NK cells secrete similar levels of cytokines following activation. Unstimulated (US) cells did not secrete any detectable amount of IFNγ (**Figure 2A**).

Next, we compared the ability of CB-NK and PB-NK cells to cause BMCA. Supernatants from US or activated CB-NK and PB-NK cells were incubated with myeloid cells (primary monocytes and THP-1). Following overnight incubation, BMCA was assessed by measuring IL-6 and IL-1β released by myeloid cells. Supernatants obtained from US cells did not cause BMCA. However, supernatants from activated PB-NK cells caused significantly higher levels of BMCA, as IL-6 (**Figure 2B**) and IL-1β (**Figure 2C**) were significantly higher in myeloid cells incubated with activated PB-NK cell supernatants compared to activated CB-NK cell supernatants. The method of NK cell activation did not affect this observation, as PB-NK cells activated by both PMA/Iono or co-culture caused significantly higher levels of BMCA compared to CB-NK cells (**Figures 2B, C**). BMCA was also assessed in other myeloid cell types. Consistent with the results obtained with primary monocytes and THP-1 cells, both primary human macrophages and THP-1-derived macrophages secreted higher levels of IL-6 and IL-1β after incubation with activated PB-NK cell supernatants compared to CB-NK cell supernatants (data not shown). This suggests that the observed difference between CB-NK and PB-NK cells in causing BMCA is not specific to one myeloid cell type.

Together, these data suggest that PB-NK cells cause significantly higher levels of BMCA and are more inflammatory than CB-NK cells. This observation is also consistent with recent clinical studies that found CB-CAR-NK cells did not cause inflammatory toxicities in patients with B cell malignancies (11, 18).

### Identification and characterization of soluble factors released by peripheral blood -derived activated NK cells that activate bystander myeloid cells

**Figure 3:**
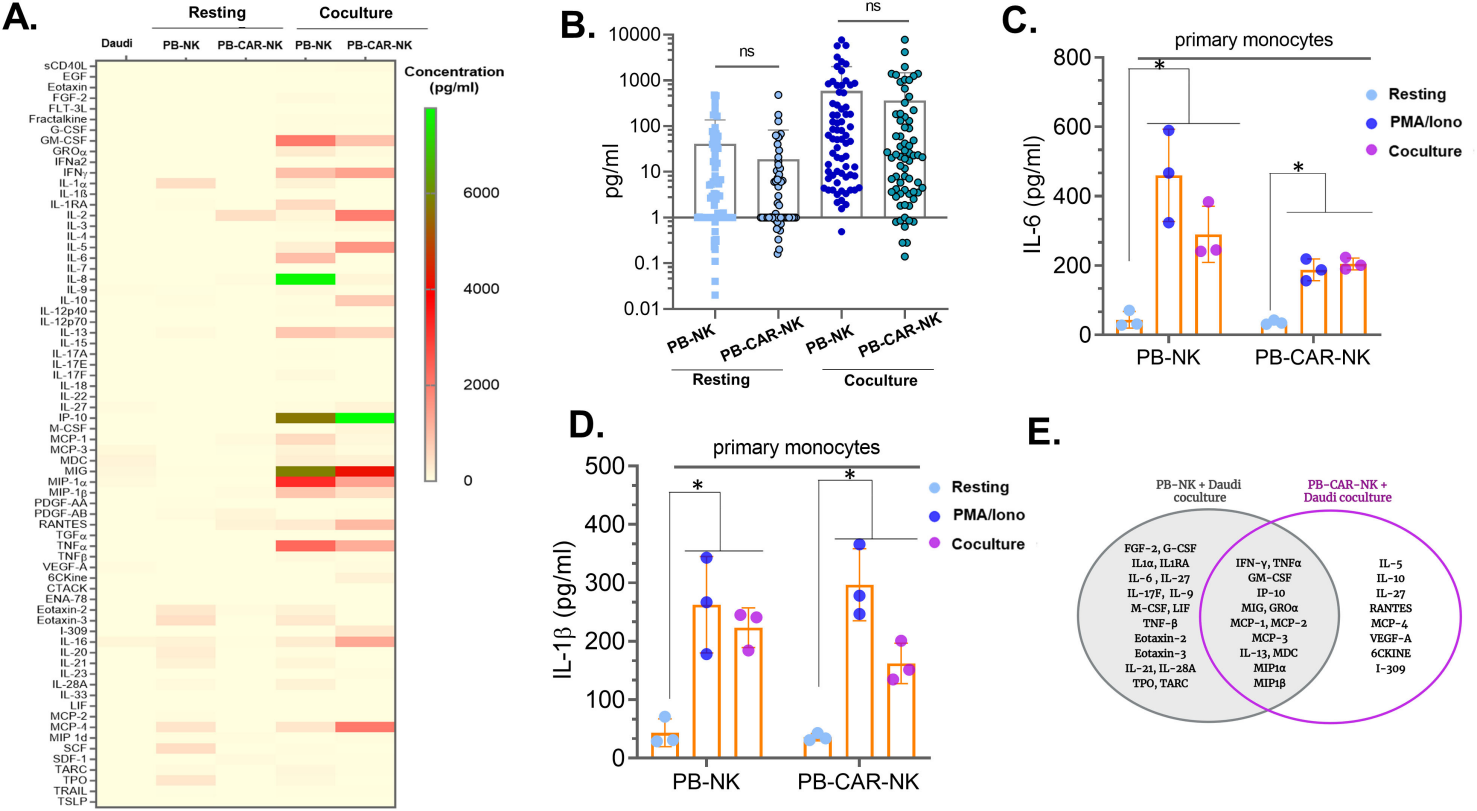
Identification and characterization of soluble factors released by peripheral blood (PB)-derived CAR-NK cells that activate myeloid cells. **(A)** Heatmap of 96 inflammatory cytokines/chemokines present in the supernatant of resting or Daudi co-culture activated PB-NK and anti-CD19 PB-CAR-NK cells (E: T= 1:1). Supernatant obtained from Daudi cells was used as a negative control. Cytokine/chemokine levels (expressed in pg/ml) are represented by a color gradient, ranging from yellow (no expression) to green (highest concentration). **(B)** Quantitation of 96 cytokines/chemokines present in the supernatants of resting or co-culture activated PB-NK and PB-CAR-NK cells. Each dot represents a cytokine or a chemokine, and average concentration obtained from three donors is shown. **(C)** IL-6 and **(D)** IL-1β secreted from primary monocytes after incubation with PB-NK and PB-CAR-NK supernatants. **(E)** Venn diagram illustrating cytokines/chemokines secreted by activated PB-NK and anti-CD19 PB-CAR-NK cells. Cytokines/chemokines unique to PB-NK cells are shown in the left circle, while those unique to PB-CAR-NK cells are shown in the right circle. The overlapping area represents cytokines/chemokines secreted by both cell types. *P<0.05, ns, not significant.

Since bystander myeloid cell activation (BMCA) was observed with both PB-CAR-NK and PB-NK cells following activation (**Figures 1**, **2**), we sought to identify and characterize the soluble factors contributing to this effect. We conducted a comparative analysis of cytokines and chemokines in supernatants obtained from activated anti-CD19 PB-CAR-NK and PB-NK cells after co-culture with CD19+ Daudi target cells (E= 1:1) for 24 hours. Supernatants from Daudi cells or resting, unstimulated PB-NK and PB-CAR-NK cells were also analyzed, revealing little to no expression of inflammatory cytokines and chemokines (**Figure 3A**). Following co-culture activation, both PB-NK and PB-CAR-NK cells secreted significantly higher levels of inflammatory cytokines and chemokines compared to the resting cells (**Figure 3A**). Although the total amount of inflammatory cytokines and chemokines released by activated PB-NK and PB-CAR-NK cells demonstrated no significant difference (**Figure 3B**), the profiles of these inflammatory cytokines and chemokines were not identical (**Figure 3A**).

Next, we assessed whether differences in the cytokine/chemokine expression profile affected the ability of PB-NK and PB-CAR-NK cells to induce BMCA. Supernatants obtained from resting, PMA/Iono activated, or co-culture activated PB-NK and PB-CAR-NK cells were incubated with primary monocytes. BMCA was assessed by measuring IL-6 and IL-1β secreted by myeloid cells. Compared to the resting cells, supernatants from both activated PB-NK and PB-CAR-NK cells significantly induced BMCA (**Figures 3C, D**). This suggests that despite differences in cytokine expression profiles, both PB-NK and PB-CAR-NK cells induce BMCA following CAR-dependent or CAR-independent activation. This led us to hypothesize that soluble factors common to both cell types contribute to BMCA.

We next assessed the soluble factors common between PB-NK and PB-CAR-NK cells following co-culture with Daudi cells. Out of 96 cytokines and chemokines evaluated, 17 were expressed only by PB-NK cells, 8 were expressed only by PB-CAR-NK cells, and 13 were expressed by both (**Figure 3E**). The remaining cytokines and chemokines were either not expressed or expressed at very low levels by both cell types following activation. All 13 cytokines/chemokines that were expressed by both PB-NK and PB-CAR-NK cells (IFNγ, GM-CSF, TNFα, IP-10, MIG, MCP-1, MCP-2, MCP-3, IL-13, GROα, MDC, MIP-1α, MIP-1β) were expressed at least 10-fold higher than unstimulated, resting cells (**Figure 3E**). These 13 common cytokines and chemokines were considered for further evaluation.

**Figure 4:**
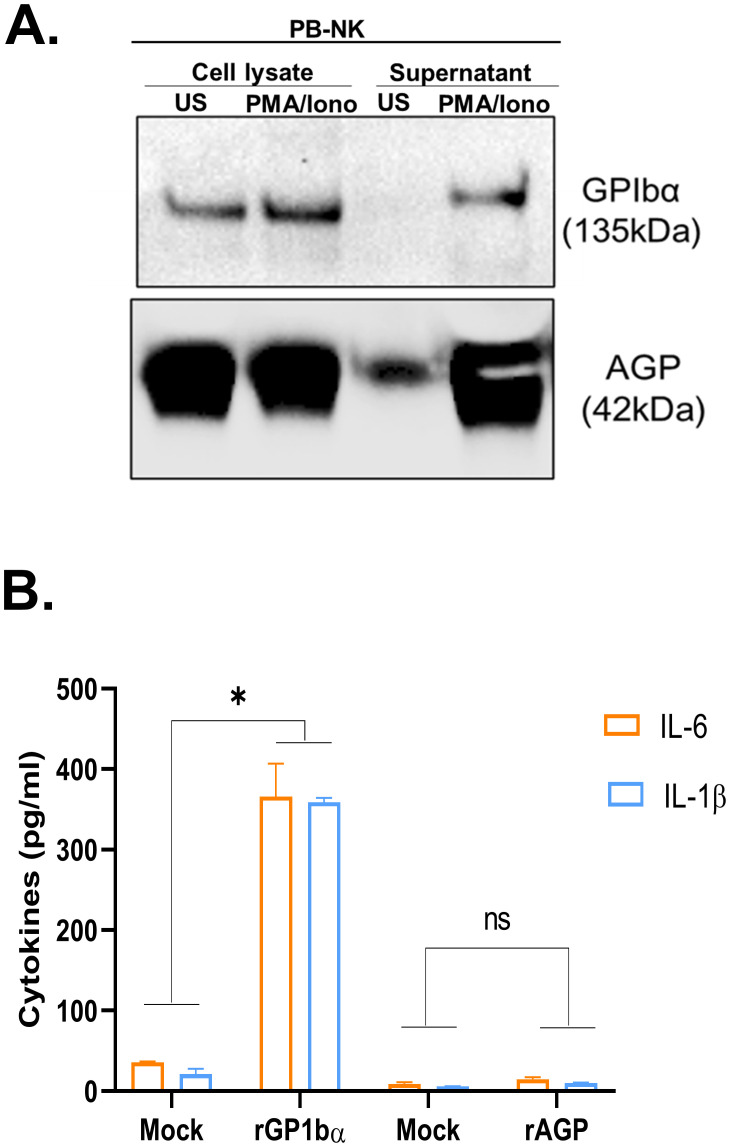
Recombinant GPIbα but not AGP causes myeloid cell activation. **(A)** Immunoblot analysis of cell lysate and supernatant obtained from unstimulated (US) or PMA/Ionomycin stimulated peripheral blood (PB) NK cells for GPIbα and AGP expression. **(B)** Recombinant proteins, rGPIbα and rAGP were added to the myeloid cells (THP-1), and IL-6 and IL-1β released in the supernatant was measured by ELISA. Mock THP-1 cells were treated with control rGFP protein. *P<0.05, ns, not significant.

In addition to the 13 cytokines and chemokines, we also evaluated two additional factors previously reported to play a role in lymphocyte-mediated inflammatory responses: glycoprotein Ib subunit alpha (GPIbα) (23) and alpha-1-acid glycoprotein (AGP) (24). Although GPIbα has primarily been studied as a receptor for von Willebrand factor (VWF) (25), a recent study found that GPIbα released by activated T cells caused monocyte activation when stimulated with muramyl dipeptide (MDP) adjuvant and recombinant GPIbα protein increased activation of MDP-treated monocytes (23). Furthermore, AGP activates bystander myeloid cells, causing the release of inflammatory cytokines, including IL-6 (24). We found that PB-NK cells express both GPIbα and AGP (**Figure 4A**). However, only after activation, PB-NK cells secrete these proteins as they were found in the supernatant from activated PB-NK cells and were absent or expressed at very low levels in the supernatant from unstimulated PB-NK cells (**Figure 4A**). This suggests that PB-NK cells secrete these proteins following activation. To confirm whether GPIbα and AGP cause myeloid cell activation, recombinant GPIbα (rGPIbα) or recombinant AGP (rAGP) were incubated with the myeloid cells (THP-1). Following overnight incubation, BMCA was assessed by measuring IL-6 and IL-1β levels. Interestingly, only rGPIbα significantly induced BMCA, while rAGP did not activate myeloid cells to release IL-6 or IL-1β (**Figure 4B**). This suggests that GPIbα, but not AGP, secreted by activated PB-NK cells contributes to myeloid cell activation.

Together, we identified a total of 14 soluble factors secreted by activated PB-NK cells (IFNγ, GM-CSF, TNFα, IP-10, MIG, MCP-1, MCP-2, MCP-3, IL-13, GROα, MIP-1α, MIP-1β, MDC, and GPIbα) with the potential to cause BMCA.

### Neutralization of select soluble factors in peripheral blood -derived CAR-NK cells reduces bystander myeloid cell activation

**Figure 5:**
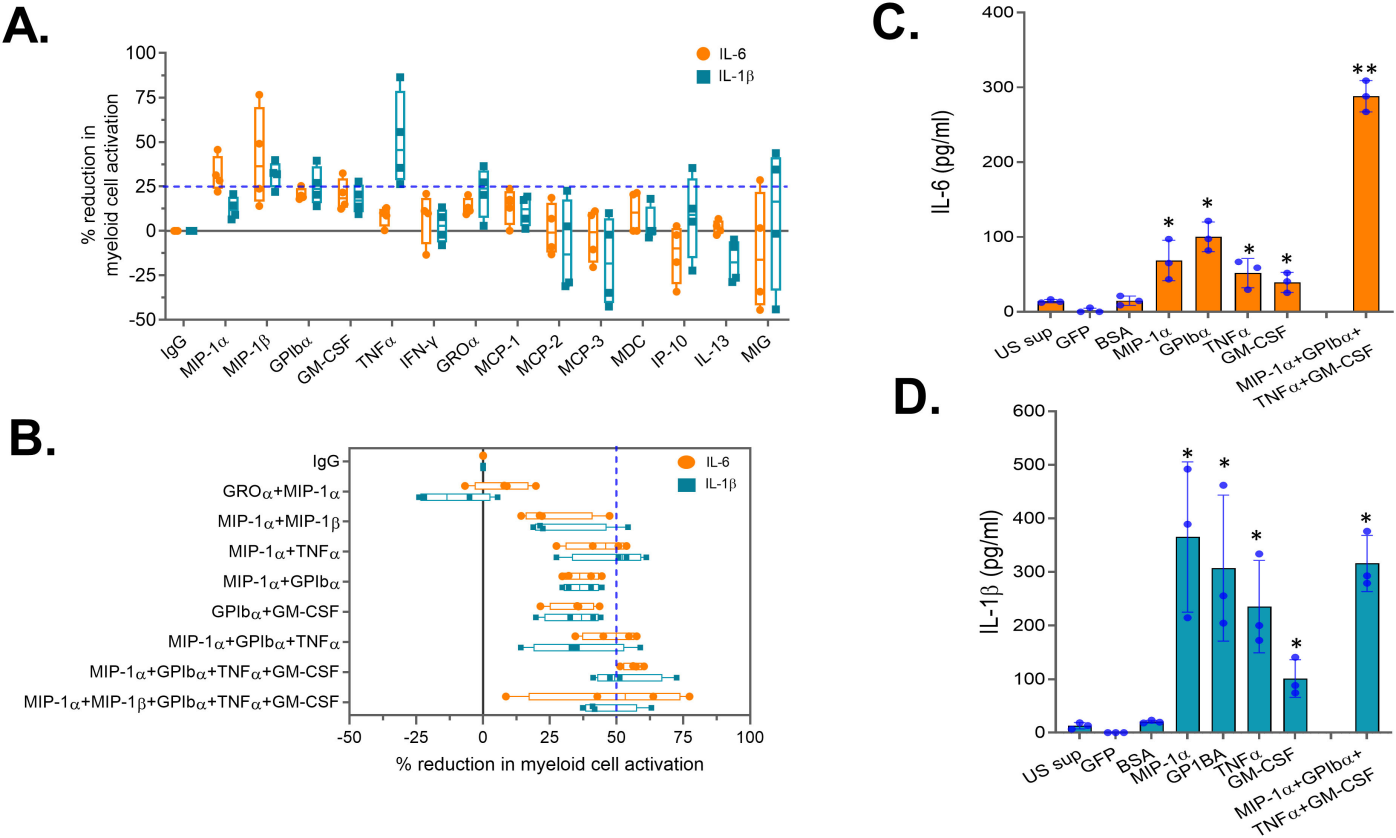
Neutralization of select soluble factors in peripheral blood (PB)-derived CAR-NK cells reduces myeloid cell activation. Supernatant obtained from activated PB-NK cells was subjected to antibody-mediated neutralization. Following neutralization, supernatant was added to the myeloid cells (THP-1) and secretion of IL-6 and IL-1β by myeloid cells were measured. Percent (%) reduction in myeloid cell activation for each antibody was calculated by normalizing to the IL-6 and IL-1β levels secreted by myeloid cells incubated with IgG control treated PB-NK supernatant. Percent (%) reduction in myeloid cell activation following neutralization of **(A)** a single or **(B)** combination of soluble factors in the PB-NK supernatant. **(C)** IL-6 and **(D)** IL-1β secreted by myeloid cells (primary monocytes) following incubation with recombinant proteins. US sup, unstimulated NK cell supernatant. *P<0.05, **P<0.01. Each dot represents one donor.

To assess the role of these 14 soluble factors in bystander myeloid cell activation (BMCA), each factor was individually neutralized in the activated supernatant obtained from PB-NK cells using factor-specific antibodies. After neutralization, the supernatant was incubated with myeloid (THP-1) cells, and BMCA was assessed by measuring IL-6 and IL-1β. Supernatant treated with non-specific IgG was used as a negative control. A percent mean reduction in myeloid cell activation (IL-6 and/or IL-1β levels) of at least 25% compared to the IgG control was considered significant. Of the 14 soluble factors, neutralization of MIP-1α, MIP-1β, GPIbα, GM-CSF, TNFα, and GROα significantly reduced BMCA, whereas neutralization of IFNγ, MCP-1/2/3, MDC, IP-10, IL-13, and MIG had minimal to no impact on BMCA (**Figure 5A**). Additionally, some factors reduced both IL-6 and IL-1β (MIP-1β, GPIbα, GM-CSF), while others reduced either IL-6 (MIP-1α) or IL-1β (TNFα, GROα) (**Figure 5A**). We also observed considerable variability in antibody-mediated neutralization between factors. For example, at the 25 µg/ml antibody concentration used in this experiment, GM-CSF was 90% neutralized, while TNFα was only 75% neutralized (**Supplementary Figure S3A**). This variability also likely contributed to the observed differences in BMCA in **Figure 5A**.

Since none of the factors’ neutralization resulted in more than a 50% reduction in IL-6 and IL-1β, we selected five factors (MIP-1α, MIP-1β, GPIbα, GM-CSF, and TNFα) whose neutralization resulted in greater than a 25% mean reduction in IL-6 or IL-1β for combination neutralization. Through the assessment of various neutralization combinations, we found that neutralizing four soluble factors—MIP-1α, GPIbα, TNFα, and GM-CSF—resulted in over a 50% mean reduction in both IL-6 and IL-1β compared to the IgG control (**Figure 5B**). Surprisingly, adding MIP-1β neutralization to the combination did not further reduce myeloid cell activation (**Figure 5B**), suggesting that BMCA is driven by select PB-NK cell factors and not all factors equally contribute to this effect.

To confirm the role of these four factors in myeloid cell activation, recombinant MIP-1α, GPIbα, TNFα, and GM-CSF were studied. All four recombinant proteins, both independently and in combination, significantly activated the myeloid (THP-1) cells (**Figures 5C, D**). Supernatant from unstimulated (US) PB-NK cells and control proteins (GFP and BSA) did not activate myeloid cells (**Figures 5C, D**). Together, these data confirm that the four soluble factors released by activated NK cells cause BMCA.

**Figure 6:**
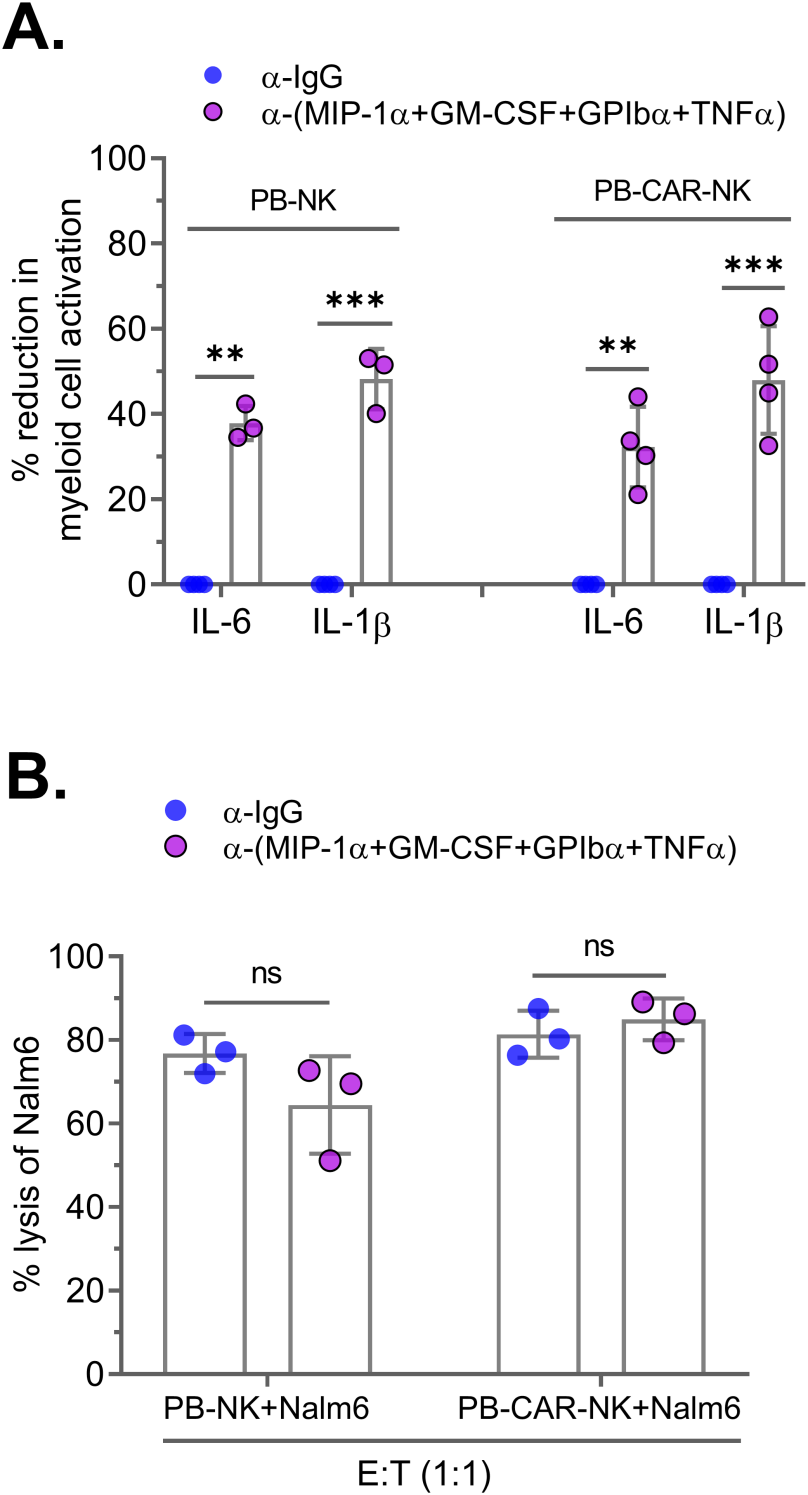
Neutralization of four soluble factors in peripheral blood (PB)-derived anti-CD19 PB-CAR-NK cells reduces myeloid cell activation without affecting CAR-NK cell potency. Supernatants obtained from Daudi co-culture (E: T= 1:1) activated PB-NK cells or anti-CD19 PB-CAR-NK cells were subjected to antibody-mediated neutralization. Following neutralization, supernatant was added to the myeloid cells (THP-1) and secretion of IL-6 and IL-1β by myeloid cells were measured. Percent (%) reduction in myeloid cell activation was calculated by normalizing to the IL-6 and IL-1β levels secreted by myeloid cells incubated with IgG control treated PB-NK or PB-CAR-NK supernatant. **(A)** Percent (%) reduction in myeloid cell activation following neutralization of GPIbα, GM-CSF, TNFα, and MIP-1α in the supernatant obtained from activated PB-NK and PB-CAR-NK cells. **(B)** NK cell potency was assessed by measuring lysis of Nalm6 target cells by PB-NK and anti-CD19 PB-CAR-NK cells in the presence of control IgG or GPIbα, GM-CSF, TNFα, and MIP-1α antibodies. **P<0.01, ***P<0.001, ns, not significant. Each dot represents one donor.

Next, we assessed the effect of neutralizing these four factors on PB-CAR-NK-mediated myeloid cell activation. PB-NK and PB-CAR-NK cells were activated following co-culture with Daudi target cells (E: T= 1:1). Supernatants obtained from the activated cells were subjected to neutralization of the four factors. After neutralization, the supernatants were incubated with myeloid (THP-1) cells, and BMCA was assessed by measuring IL-6 and IL-1β. We found that BMCA was significantly reduced in both PB-NK and PB-CAR-NK cells following neutralization of the four factors (**Figure 6A**). It is important to note that antibody-mediated combination neutralization of these four factors does not result in a 100% reduction in their expression (**Supplementary Figure S3B**). For example, at a total antibody concentration of 50 µg/ml (12.5 µg/ml per cytokine), we observed approximately a 60% reduction in cytokine levels following combination neutralization. The residual cytokines present in the antibody-treated supernatant may still contribute to myeloid cell activation, explaining the inability to achieve a complete reduction in myeloid cell activation (**Figure 6A**).

We also assessed the impact of neutralizing these four factors on NK cell potency. Both anti-CD19 PB-CAR-NK and PB-NK cells were co-cultured with CD19+ Nalm6 target cells (E: T = 1:1) in the presence of four factor-specific antibodies or an IgG control, and cytotoxicity was measured after 24 hours. Neutralization of the four factors did not affect the cytotoxic function of either PB-CAR-NK or PB-NK cells (**Figure 6B**).

Since NK-92 is a clinically relevant cell line that has also been used in clinical trials to manufacture CAR-NK cells (13), we assessed whether neutralization of the four factors in supernatant obtained from activated NK-92 cells also reduces myeloid cell activation. Consistent with the results obtained in PB-NK and PB-CAR-NK cells, neutralization of the four factors in activated NK-92 cell supernatant significantly reduced BMCA (**Supplementary Figure S4A**) without affecting its potency (**Supplementary Figure S4B**).

Together, these data suggest that neutralization of four factors—MIP-1α, GPIbα, TNFα, and GM-CSF—significantly reduces PB-NK, PB-CAR-NK, and NK-92 cell-mediated BMCA without affecting their function.

### siRNA-mediated knockdown of inflammatory factors in peripheral blood -derived CAR-NK cells reduces bystander myeloid cell activation

**Figure 7:**
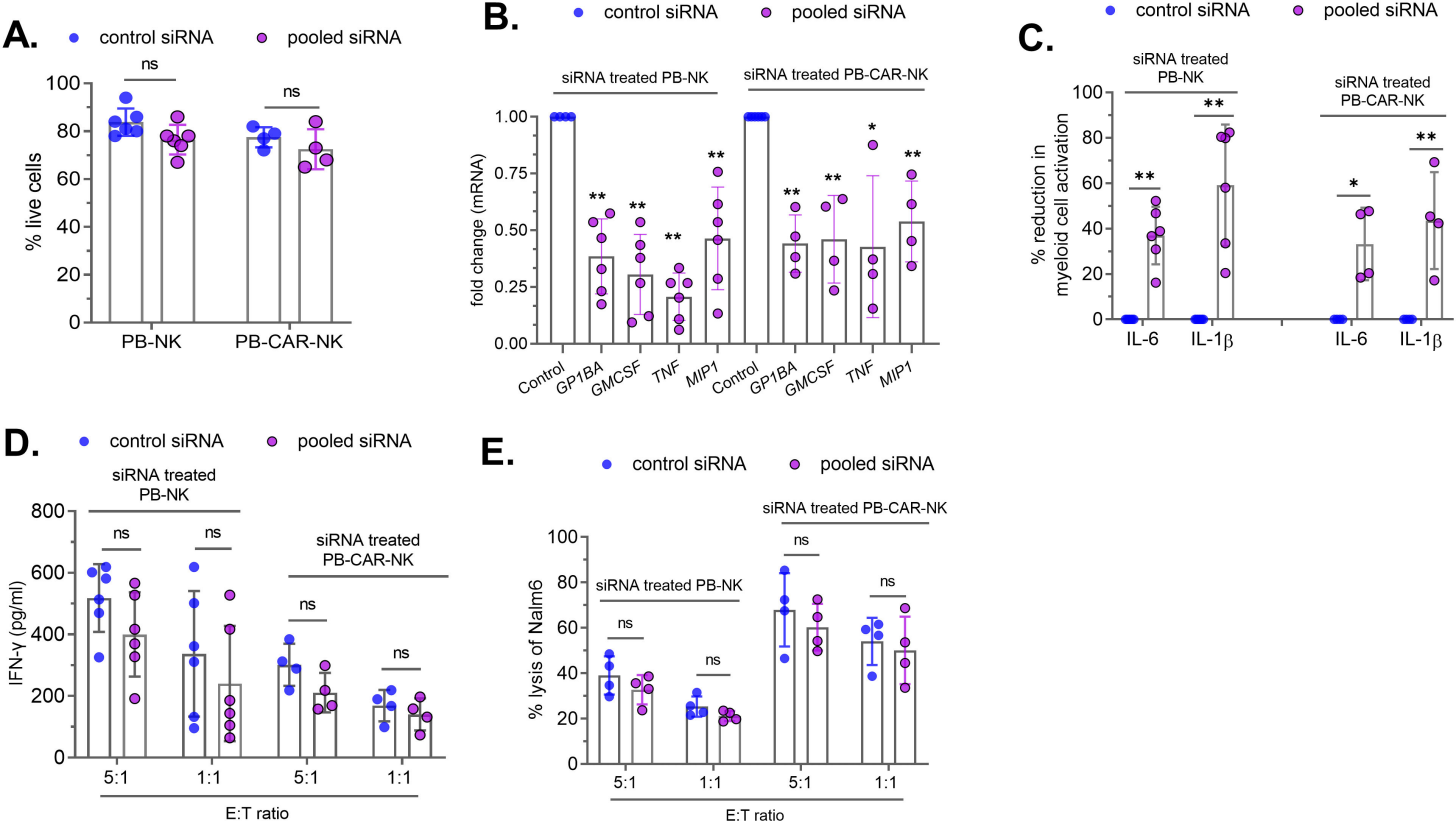
siRNA-mediated knockdown of four soluble factors in peripheral blood (PB)-derived PB-NK and PB-CAR-NK cells reduces myeloid cell activation. PB-NK or anti-CD19 PB-CAR-NK cells were transfected with the control siRNA or the pooled siRNAs to reduce GPIbα, GM-CSF, TNFα and MIP-1α protein expression. **(A)** Cellular viability and **(B)** mRNA were measured after 48 hours of siRNA transfection. siRNA-treated PB-NK or anti-CD19 PB-CAR-NK cells were activated following co-culture with Daudi target cells (E: T =1:1). Myeloid (THP-1) cells were incubated with supernatants from siRNA-transfected and activated PB-NK and PB-CAR-NK cells, and IL-6 and IL-1β secreted by myeloid cells were measured. **(C)** Percent (%) reduction in myeloid cell activation was calculated by normalizing to the IL-6 and IL-1β levels secreted by myeloid cells incubated with control siRNA treated PB-NK or PB-CAR-NK cell supernatant. siRNA-transfected PB-NK or PB-CAR-NK cells were co-cultured with Daudi cells at an effector to target (E: T) ratios of 5:1 and 1:1 for 24 hours. **(D)** IFNγ secreted by PB-NK and PB-CAR-NK cells following co-culture was measured. **(E)** NK cell potency was assessed by measuring lysis of Nalm6 target cells by PB-NK and anti-CD19 PB-CAR-NK cells following siRNA treatment. *P<0.01, **P<0.00,1 ns, not significant. Each dot represents one donor.

The identification of four soluble factors released by PB-CAR-NK cells that cause bystander myeloid cell activation (BMCA) presents an opportunity to genetically modify PB-derived CAR-NK cells during manufacturing to improve product safety. To assess whether the knockdown of four inflammatory factors (MIP-1α, GPIbα, TNFα, and GM-CSF) in NK cells reduces CAR-NK-mediated BMCA, PB-NK and PB-CAR-NK cells were transfected with a pool of four different siRNAs targeting each of these factors (pooled siRNA) or control non-specific siRNA (Control siRNA) for 48 hours. siRNA transfection did not affect the cellular viability of PB-NK or PB-CAR-NK cells (**Figure 7A**). As observed with antibody-mediated neutralization, siRNA-mediated knockdown did not completely reduce the expression of these inflammatory factors at both the mRNA (**Figure 7B**) and the protein (**Supplementary Figure S5**) levels. GPIbα protein expression was not assessed due to the unavailability of an assay to quantify NK cell secreted GPIbα levels.

Next, siRNA-treated cells were activated by co-culturing with Daudi (target) cells at an effector-to-target (E: T) ratio of 1:1, and cell-free supernatants were harvested after 24 hours. The supernatants obtained from both siRNA-treated PB-NK and PB-CAR-NK cells were incubated with myeloid (THP-1) cells, and BMCA was assessed by measuring IL-6 and IL-1β. We found that siRNA-mediated knockdown of the four factors significantly reduced BMCA (**Figure 7C**). As observed with antibody-mediated neutralization (**Supplementary Figure S3B**), siRNA treatment did not completely reduce the expression of these inflammatory factors (**Figure 7B**; **Supplementary Figure S5**). The residual levels of these inflammatory proteins in siRNA-treated cells may contribute to BMCA, potentially explaining the incomplete reduction of BMCA.

To assess whether the knockdown of these four factors affected CAR-NK cell potency, siRNA-treated PB-NK or anti-CD19 PB-CAR-NK cells were co-cultured with CD19+ Nalm6 (target) cells at E:T ratios of 5:1 and 1:1. Following 24 hours of co-culture, IFNγ release and cytotoxicity were assessed. There was no significant difference in IFNγ production by PB-NK and PB-CAR-NK cells treated with siRNA against the four factors (pooled siRNA) compared to control siRNA (**Figure 7D**). Similarly, PB-NK and PB-CAR-NK cell-mediated cytotoxicity was also not affected by the knockdown of these four factors (**Figure 7E**).

Together, these data demonstrate that the knockdown of MIP-1α, GPIbα, TNFα, and GM-CSF in PB-CAR-NK cells reduces myeloid cell activation without affecting their function.

## Discussion

CAR-T cell therapy is associated with life-threatening inflammatory toxicities in part due to secondary activation of bystander immune cells (e.g., myeloid cells) following primary activation of CAR-T cells (1, 16). Limited clinical studies suggest that CAR-NK cells-derived from cord blood (CB) may be safer than peripheral blood (PB)-derived CAR-T cells (11, 18). However, CAR-NK cells derived from other sources have caused inflammatory toxicities in clinical and nonclinical studies (12, 13). Furthermore, prior studies have not comprehensively assessed the inflammatory potential of NK cells obtained from various sources. Given the data on inflammatory toxicities during CAR-NK cell therapy is limited, it is important to understand whether T cells are more inflammatory than NK cells and if the source of NK cells affects their potential to cause inflammatory toxicities.

Like T cells, NK cells secrete a myriad of immunoregulatory cytokines following activation. Some of these cytokines are essential for NK cell function, while others may contribute to unwanted side effects. In this study, we found that peripheral blood (PB)-derived CAR-T cells cause significantly higher bystander myeloid cell activation compared to PB-CAR-NK cells, suggesting that PB-CAR-T cells are more inflammatory than PB-CAR-NK cells. Although PB-CAR-NK cells caused significantly less myeloid cell activation than PB-CAR-T cells, it was not absent and considerable myeloid cell activation was observed with PB-CAR-NK cells, indicating that PB-NK cells are capable of inducing myeloid cell activation. Interestingly, NK cells from cord blood (CB) did not cause the same level of myeloid cell activation observed with PB-NK cells. These data suggest that PB-T cells are more inflammatory than PB-NK cells, and PB-NK cells are more inflammatory than CB-NK cells. These findings are consistent with published clinical studies suggesting that CB-NK cells have a lower inflammatory potential and may be safer (18, 26).

To characterize the inflammatory cytokines secreted by PB-derived CAR-T cells and PB-NK cells, both cell types were co-cultured with target cells, and the resulting supernatant was analyzed using inflammatory cytokine arrays. We found that PB-CAR-T cells secreted higher levels of inflammatory cytokines than PB-CAR-NK cells following activation. Notably, the primary cytokines associated with CRS and inflammation were at least 10-fold higher in PB-CAR-T cells compared to PB-CAR-NK cells. This observation aligns with clinical findings in CAR-T cell therapy, where elevated cytokine profiles are observed in patients experiencing inflammatory toxicities.

Additionally, intra-donor comparative analysis revealed that activated PB-T cells secrete significantly higher levels of inflammatory cytokines than activated PB-NK cells from the same donor. These elevated cytokine levels in activated T cells may contribute to the more frequent cases of inflammatory toxicities observed in clinical studies involving CAR-T cell therapy. While our results suggest that CAR-NK cells may have a comparatively lower potential to cause inflammatory toxicities, it is important to note that activated PB-CAR-NK cells did cause significant myeloid cell activation compared to resting PB-CAR-NK cells. Both CAR-dependent and CAR-independent activation of PB-NK cells caused myeloid cell activation, suggesting that factors secreted by NK cells, independent of CAR signaling, drive this effect and soluble factors common between CAR-dependent and CAR-independent activation of NK cells may contribute to myeloid cell activation.

To identify these common factors, supernatants from activated PB-NK and PB-CAR-NK cells were assessed using cytokine arrays. Two additional factors, AGP and GPIbα, not included in the cytokine array, were selected due to their known role in causing inflammation. AGP is a well-known inflammatory protein that activates myeloid cells to produce IL-6 (24), while T cell-derived GPIbα induces myeloid activation via Mac-1 signaling (23). We found that both AGP and GPIbα were secreted by PB-NK cells following activation; however, only recombinant GPIbα, and not AGP, caused myeloid cell activation. and alpha-1-acid glycoprotein (AGP). Thus, a total of 14 soluble factors (IFNγ, GM-CSF, GPIbα, TNFα, IP-10, MIG, MCP-1, MCP-2, MCP-3, IL-13, GROα, MDC, MIP-1α, MIP-1β) were identified as common factors between PB-NK and PB-CAR-NK cells with the potential to induce myeloid cell activation. Notably, IFNγ and GM-CSF have previously been identified as contributors to inflammatory toxicities in CAR-T cell therapy (27–29).

The role of each of the identified factor in myeloid cell activation was then assessed through antibody-based neutralization studies, which identified four factors—GM-CSF, GPIbα, TNFα, and MIP-1α—as primary contributors to PB-NK and PB-CAR-NK-mediated myeloid cell activation, with neutralization of these factors resulting in at least a 50% reduction in myeloid cell activation. Furthermore, recombinant GM-CSF, GPIbα, TNFα, and MIP-1α also activated myeloid cells, confirming their role in mediating inflammatory toxicities. Importantly, neutralization of these four factors did not affect PB-CAR-NK cell-mediated cytotoxicity, suggesting that while these factors contribute to inflammatory toxicities, they are not required for PB-CAR-NK cell function.

As noted above, identifying key inflammatory factors in NK cells offers a potential avenue to enhance the safety of CAR-NK cell products by targeting these factors during manufacturing. To explore this prospect, we conducted siRNA-mediated knockdown experiments targeting the four identified factors in PB-NK and PB-CAR-NK cells. While we observed variability in the silencing efficiency across these factors, ranging from 20% to 80%, the collective knockdown significantly reduced myeloid activation without compromising the potency of PB-NK and PB-CAR-NK cells. This underscores the feasibility of modulating specific inflammatory factors to improve the safety profile of CAR-NK cell products during manufacture. In both the antibody-mediated neutralization and siRNA-mediated knockdown studies, the incomplete reduction in expression of these four factors suggests that residual levels likely contributed to myeloid cell activation. Additionally, other factors secreted by activated PB-NK cells may also contribute to myeloid cell activation.

**Figure 8:**
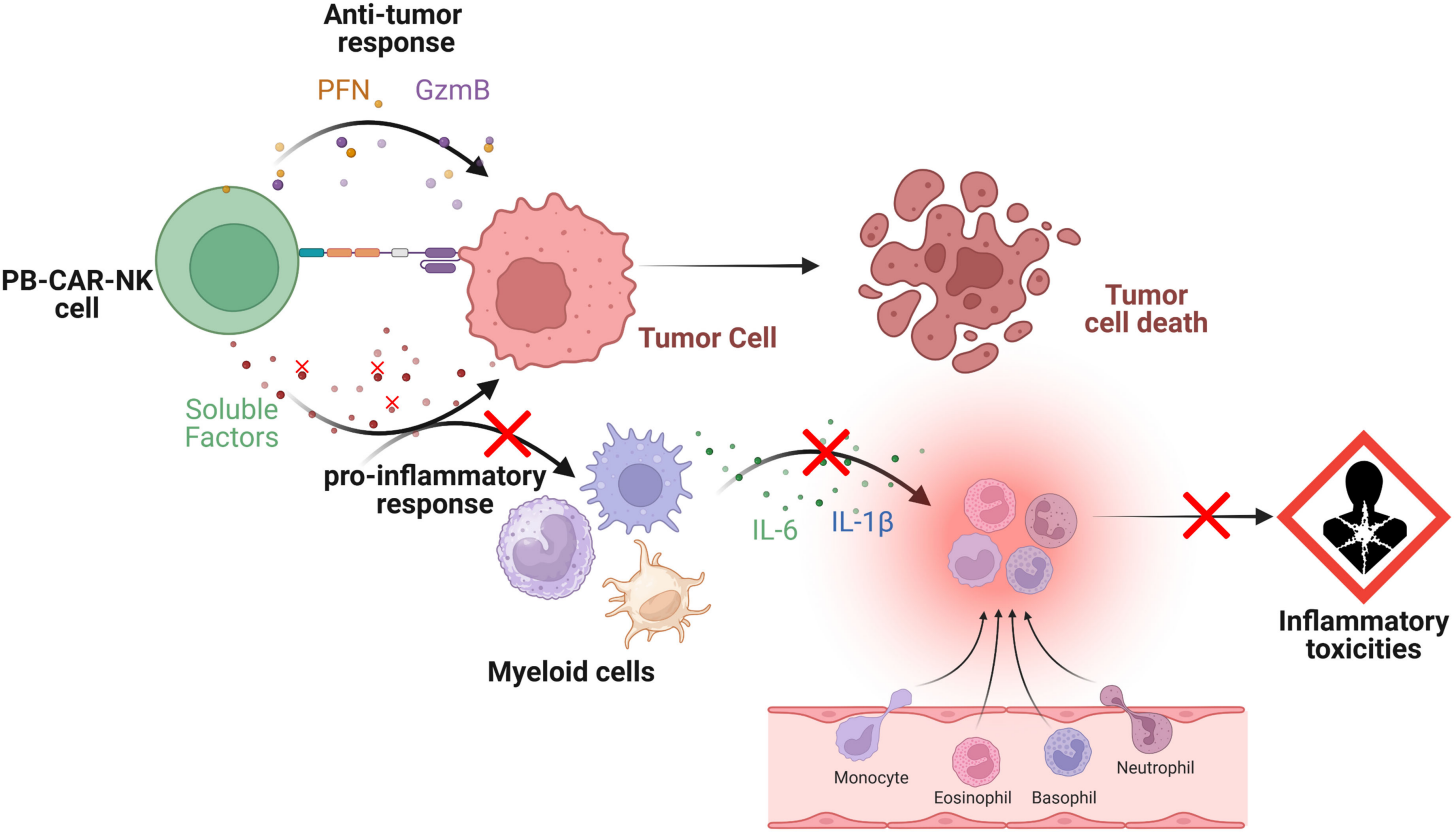
Schematic representation of the interactions between peripheral blood (PB)-derived CAR-NK cells, target tumor cells and bystander myeloid cells. Upon engagement, PB-CAR-NK cells become activated and secrete various soluble factors (e.g., perforin [PFN], granzyme B [GzmB], cytokines, and chemokines), which are critical for their effector functions. However, some of these secreted factors also induce the activation of bystander immune cells, such as monocytes and macrophages, contributing to the pro-inflammatory response. Once activated, these bystander myeloid cells release pro-inflammatory mediators, including IL-6 and IL-1β, which contribute to inflammatory toxicities. Depletion or inactivation of specific soluble factors in PB-CAR-NK cells (shown by red crosses) that contributes to the pro-inflammatory response but not effector functions (e.g., anti-tumor response) reduces bystander myeloid cell activation, leading to lower inflammatory toxicities (shown by red crosses). Figure created with BioRender.com.

Overall, our study suggests that both PB-CAR-T and PB-CAR-NK cells can activate bystander myeloid cells and have the potential to cause inflammatory toxicities during CAR-based therapies. We also found that CB-NK cells are significantly less inflammatory than PB-NK cells. Furthermore, we identified four soluble factors released by PB-CAR-NK cells which, upon neutralization or knockdown, significantly reduced myeloid cell activation without affecting CAR-NK cell function. This suggests that inactivating these factors during CAR-NK cell manufacture may improve product safety (**Figure 8**).

Since this study focused on inflammatory cytokines and chemokines included in the cytokine array, additional soluble factors secreted from PB-NK cells may also contribute to myeloid cell activation. Moreover, the mechanisms behind the lower inflammatory response of CB-NK cells remain poorly understood. Current studies are underway to address these knowledge gaps. Understanding the mechanisms contributing to inflammatory toxicities during CAR-NK cell therapy can help improve the safety and efficacy of these therapies.

## Materials and methods

### Isolation of primary cells

Buffy coats from healthy donors were obtained from the National Institutes of Health (NIH) Blood Bank. All donors provided written consent for their blood products to be used in research projects and all blood samples were deidentified. This study was exempted by the FDA’s IRB, Research Involving Human Subject Committee (RIHSC). PBMCs were isolated using Ficoll gradient centrifugation, washed in 1X PBS and resuspended in complete RPMI 1640 media supplemented with 2mM L-glutamine, 100 IU/mL penicillin, 10 μg/mL streptomycin and 10% heat-inactivated FBS, and incubated overnight at 37°C, 5% CO2. The following day, cells in suspension were used to isolate the NK and T cells using cell type-specific isolation kits (Mitleyni Biotec). Cell purity was assessed by flow cytometry by staining for T cells (CD3-PE; Biolegend 317308), and NK cells (CD56-APC; BD 341025). We routinely obtained over 85% pure cells in our experiments (**Supplementary Figure S6**). Adherent monocytes were washed 3 times with 1X PBS and treated with trypsin for 3 minutes at 37°C. Trypsin was neutralized with complete RPMI media and cells were counted. For donor-matched experiments, NK, T cells and primary monocytes were isolated from the same donor. Primary NK cells were cultured in NK MACs media (Miltenyi Biotec) according to manufacturer’s instructions, primary monocytes were cultured in complete IMDM media, and primary T cells were cultured in complete RPMI. Human umbilical cord blood mononuclear cells (MNCs) were purchased from STEMCELL Technologies. Frozen CB MNCs from different donors were obtained, and NK cells were isolated using NK cell Isolation kit (Miltenyi Biotec) and cultured in the NK media as described above.

### Cell lines

THP-1, Daudi and NK-92 cells were purchased from ATCC and HEK 293T cells from Cell Genesys. All cells were maintained at 37°C with 5% CO2. THP-1 cells were cultured in IMDM media (ATCC) supplemented with 100 IU/ml penicillin, 10µg/ml streptomycin, and 10% heat-inactivated fetal bovine serum (HI-FBS; Atlanta Biologicals). NK-92 cells were cultured in Essential Medium supplemented with 100 IU/ml penicillin, 10µg/ml streptomycin, 2mM GlutaMax (Thermo), 10% horse serum and 10% FBS (not heat-inactivated). HEK293T cells were cultured in DMEM media (Lonza) supplemented with 100 IU/ml penicillin, 10µg/ml streptomycin, 2mM GlutaMax (Thermo), and 10% heat-inactivated FBS. Nalm6 expressing GFP/luciferase with beta-2 microglobin (B2M) knock out were previously described (30). Daudi cells were cultured in RPMI 1640 with 100 IU/ml penicillin, 10µg/ml streptomycin, 2mM GlutaMax (Thermo) and 10% HI-FBS.

### THP-1 macrophages and primary macrophages

THP-1 cell line and PB-derived primary monocytes were used to differentiate into THP-1 macrophages and primary macrophages using the published protocol and differentiation was assessed by microscopy as well as secretion of TNFα in the supernatants (31). Over 90% differentiation was achieved in our experiments.

### Lentiviral vector

Production of lentiviral vector encoding anti-CD19 CAR (anti-CD19 scFv-CD28-41BB-CD3z) has been described previously (30). Briefly, plasmids encoding VSV-G envelope, CD19 CAR and packaging plasmid pCD/NL-BH*DDD were mixed with PEI-Max (Polysciences) in Opti-MEM media (Gibco) for 15 minutes and added to HEK293T cells for 6 hours. Cells were washed with 1X PBS and resuspended in complete media for 72 hours at 37°C. Supernatant was filtered using 0.4µm filter (VWR) and concentrated using a 100kDa filter (Millipore Sigma). Concentrated supernatant with lentivirus vector was aliquoted, stored at -80°C. Infectious titer was determined using Jurkat cells and NK-92 cells by adding serially diluted vector in polybrene (AmericanBio) to 1x10^6^ Jurkat or NK-92 cells for 72 hours. CAR expression on live cells was measured by flow cytometry (BD LSR Fortessa) using PE conjugated Protein L (AcroBio). Vector titer was calculated as T = (P*N)/(D*V), where T = titer (TU/ml); P = % PE positive cells, N = number of cells at the time of transduction, D = dilution factor and V = total volume of viral inoculum.

### PB-CAR-NK cells and PB-CAR-T cells

Primary NK and T cells isolated from PBMCs were activated. NK cells were activated following co-culture with Daudi cells, and T cells were activated using anti-CD3/CD28. Following 48 hours of activation, cells were transduced with lentiviral vector at MOI of 10. Cells were subjected to spinoculation at 1,800 rpm for 60 minutes at 25°C in 6-well non-tissue culture treated and retronectin coated plate. Cells were incubated at 37°C for at least 6 hours, media was exchanged, and cells were expanded for 14 days. Untransduced cells also underwent mock transduction and expansion. Cells were monitored daily, and cell density was maintained at 0.5×10^6^ cells/mL. Following 14 days of expansion, PB-CAR-T and PB-CAR-NK cells were harvested, washed twice in 1X PBS and cryopreserved in cell freezing media (90% FBS, 10% DMSO). CAR-expressing cells were assessed for viability, CD19-CAR expression (Protein L), endotoxin (ToxinSensor Chromogenic LAL Endotoxin Assay Kit, Genscript) and cytotoxicity before using in the experiments. The CAR % was routinely found between 22-46% in CAR-NK cells and 40-68% in CAR-T cells respectively. All anti-CD19 CAR cells produced from peripheral blood (PB) were labelled as PB-CAR-T and PB-CAR-NK throughout the study.

### Activation of NK and T cells for myeloid cell activation

Peripheral blood derived primary T (PB-T), peripheral blood-derived primary NK (PB-NK), PB-derived CAR-T (PB-CAR-T), PB-derived CAR-NK (PB-CAR-NK), cord blood-derived NK (CB-NK) and NK-92 cells were activated using PMA/Ionomycin or were co-cultured with target cells (Daudi) for 24 hours at 37C. Following activation, supernatant was collected and centrifuged at 2,000xg for 10 minutes to obtain cell-free supernatant.

### Myeloid cell activation assay

Primary human monocytes, primary macrophages, THP-1 cells and THP-1 derived macrophages were used to assess myeloid cell activation. Supernatants from either unstimulated (US) or activated PB-NK, PB-T, CB-NK, PB-CAR-NK, PB-CAR-T and NK-92 cells were added to different myeloid cells (1x10^6^ cells/ml) and incubated for 24 hours. Myeloid cell activation (MCA) was assessed by measuring IL-6 and IL-1β released in the supernatant by ELISA. Percentage reduction in myeloid cell activation (MCA) was calculated normalizing and comparing the levels of IL-6 and IL-1β against respective IgG controls.

### Cell count and viability

Cell count and viability were determined using automated cell counter (Countess II, ThermoFisher).

### ELISA

IFNγ, GM-CSF, TNFα, IL-6 and IL-1β were quantified using ELISA kits from BD Biosciences, and MIP-1α ELISA kit was purchased from Thermo Scientific and used according to manufacturer’s instructions. ID5 (Molecular Devices) or Varioskan Lux (Thermo Scientific) plate readers were used to read ELISA plates.

### Cytokine array

Cell-free supernatants from PB-CAR-T, PB-CAR-NK, PB-NK and NK-92 cells were assessed for inflammatory cytokines by Eve Technologies, Canada using the Human Cytokine/Chemokine Assay.

### Antibody-mediated neutralization

Antibodies against the following proteins were used in the neutralization studies and purchased from R&D Systems: GPIbα, IFNγ, GM-CSF, AGP, TNFα, IP-10, MIG, MCP-1, MCP-2, MCP-3, IL-13, GROα, MIP-1α, MIP-1 β and MDC. Non-specific IgG was used as a control. IgG control antibody was purchased from SantaCruz. Optimization of antibody concentration for neutralization studies was performed using anti-GM-CSF and anti-TNFα antibody in primary NK and NK-92 cells. Antibody concentration of 25 µg/ml was found to be optimum. For neutralization, antibody was added to activated cell-free supernatants and mixed for 2 hours at room temperature. For combination neutralization, each antibody concentration was equally divided, and that total concentration of antibodies did not exceed 50 µg/ml. Equal amount of IgG control was used for each neutralization. Following neutralization, myeloid activation was assessed as described above.

### Recombinant proteins

Recombinant proteins GPIbα and GM-CSF were purchased from R&D Systems and TNFα, MIP-1α and MIP-1β were purchased from Acro Biosciences. Recombinant GFP (BPS BioSciences) and BSA were used as control proteins. Myeloid cells (THP-1 or primary monocytes) cells were incubated with the recombinant proteins (25 µg/ml) in the presence of resting NK cell supernatant for 24 hours, and myeloid cell activation was assessed as described above.

### Cytotoxicity assay

Cytotoxicity assay was performed as previously described (31). Briefly, CD19+ Nalm6 cells (target cells) expressing firefly luciferase were co-cultured with PB-NK or PB-CAR-NK cells (effector cells) at various effector to target ratios (E: T) in a 96-well plate. For example, 5000 target cells were co-cultured with either 50000, 25000 or 5000 effector cells. As a negative control Nalm6 cells were plated alone, and as a positive control, Nalm6 cells were treated with 10% Triton-X. Following 24 hours, an equal volume of Bright Glo luciferase reagent (100 μL) (Promega) was added to the wells and luminescence was measured using Varioskan plate reader (Thermo Scientific) in a white walled 96-well plate. Cytotoxicity was calculated as Cytotoxicity (% lysis) =100− [(Luminescence for co-culture sample/Luminesce for Nalm6 alone) ×100].

### siRNA and gene expression analysis

Non-specific control siRNA and gene specific siRNAs (*GP1BA*, *GMCSF*, *TNF* and *MIP1)* were purchased from Thermo Fischer. Primers (*B2M*, *GP1BA*, *GMCSF*, *TNF* and *MIP1)* for qPCR were purchased from Origene. Concentration of siRNAs for transfection and primers for qPCR were optimized before use following previously described procedures (32, 33). NK cells were transfected with siRNAs using Amaxa nucleofector system (Lonza) according to manufacturer’s protocol. Human Natural Killer Cell Nucleofector kit (Lonza) was used for transfection. Following 48 hours of siRNA transfection, mRNA and protein expression was assessed. For mRNA expression, total cellular RNA was extracted using RNeasy Mini Kit (Qiagen) following DNase treatment (RNase-Free DNase Set, Qiagen). Complementary DNA (cDNA) was generated using iScript cDNA kit (Bio-Rad) and relative expression determined and normalized to beta-2 microglobin expression using CFX96 Real-time PCR detection system (Biorad). For protein expression, siRNA transfected cells were activated for 24 hours, and supernatant was assessed by ELISA.

### Flow cytometry

Cells were incubated with respective antibodies for 1 hour and were subsequently washed 2x with PBS. Data was acquired on BD LSR Fortessa using single stained cells for compensation. At least 10,000 total events were collected and FlowJo program (Tree Star Inc.) was used for data analysis.

### Immunoblot analysis

Cellular lysates obtained from PB-NK activated cells were mixed with Laemmli sample buffer, heated at 95 °C for 5 min and separated on NuPAGE Bis-Tris gels by electrophoresis and transferred to nitrocellulose membranes using the iBlot transfer system (Thermo Scientific). Membranes were incubated in previously heated 3% fat-free dry milk for 1 h at room temperature, followed by overnight incubation with primary antibodies. Proteins were detected with Super Signal West Dura (Thermo Scientific) using a Bio-Rad ChemiDoc MP imaging system. Primary antibodies used were anti-GPIbα and anti- AGP from R&D Systems.

### Statistics

One-way analysis of variance was used to compare results from multiple groups and a two-sided Student’s t test was used to compare results from two groups. P values <0.05 were considered statistically significant. GraphPad PRISM (GraphPad Software Inc.) was used for statistical analysis.

## Data Availability

The original contributions presented in the study are included in the article/**Supplementary Material**. Further inquiries can be directed to the corresponding author.

## Supplementary material

Supplementary Figure 1 Myeloid cell activation following CAR-independent activation of peripheral blood (PB)-derived NK (PB-NK) and T (PB-T) cells. **(A)** Heatmap of 96 inflammatory cytokines/chemokines present in the supernatant of resting or co-culture activated anti-CD19 PB-CAR-T cells and anti-CD19 PB-CAR-NK cells (E: T= 1:1). Cytokine/chemokine levels (expressed in pg/ml) are represented by a color gradient, ranging from yellow (no expression) to green (highest concentration). **(B)** IFNγ and **(C)** GM-CSF secreted by PB-NK cells and PB-T cells following CAR-independent activation using PMA/Ionomycin. Myeloid cell activation by activated PB-T and PB-NK cells was assessed by incubating supernatant from panel B with **(D)** donor-matched primary monocytes or **(E)** THP-1 cells and measuring IL-6 and IL-1 β released by myeloid cells. *P<0.05, **P<0.01. Each dot represents one donor, and the line represents cells from the same donor.

Supplementary Figure 2 Myeloid cells secrete IL-6 and IL-1β following stimulation with activated NK cell supernatant. IL-6 and IL-1β was measured in cell culture media, human AB serum used in cell culture and various cell types following PMA/Ionomycin stimulation or in THP-1 cells following stimulation with activated NK cell supernatant. None of the cells produced significant amount of IL-6 and IL-1β following PMA/Ionomycin stimulation suggesting these cells are not the major producer of these cytokines. Importantly, THP-1 cells also did not produce IL-6 and IL-1β following PMA/Ionomycin stimulation and IL-6 and IL-1β was only detected following stimulation of THP-1 cells with activated PB-NK cell supernatant.

Supplementary Figure 3 Antibody-mediated neutralization of cytokines. **(A)** Activated PB-NK cell supernatants were incubated with either GM-CSF or TNFα antibodies at two different concentrations, 10 and 25 µg/ml. Efficiency of neutralization was assessed by measuring these cytokines in the neutralized supernatant by ELISA. **(B)** Activated PB-NK cell supernatant was incubated with pooled antibodies (GM-CSF, TNFα and MIP-1α) at 12.5µg/ml for each antibody. Efficiency of neutralization was assessed by measuring these cytokines in the neutralized supernatant by ELISA. Cytokine levels in the antibody neutralized supernatant was normalized to the levels present in the control IgG neutralized supernatant. Each dot represents one donor.

Supplementary Figure 4 Neutralization of four soluble factors in NK-92 cells reduces myeloid cell activation without affecting NK-92 cell potency. Supernatant obtained from Daudi co-culture (E: T= 1:1) activated NK-92 cells was subjected to antibody-mediated neutralization. Following neutralization, supernatant was added to the myeloid cells (THP-1) and secretion of IL-6 and IL-1β by myeloid cells were measured. Percent (%) reduction in myeloid cell activation was calculated by normalizing to the IL-6 and IL-1β secreted by myeloid cells incubated with IgG control treated NK-92 supernatant. **(A)** Percent (%) reduction in myeloid cell activation following neutralization of GPIbα, GM-CSF, TNFα, and MIP-1α in the supernatant obtained from activated NK-92 cells. **(B)** NK-92 cell potency was assessed by measuring lysis of Nalm6 target cells by NK-92 cells in the presence of control IgG or GPIbα, GM-CSF, TNFα, and MIP-1α antibodies. ***P<0.001, ns= not significant. Each dot represents one independent experiment.

Supplementary Figure 5 Protein expression following siRNA-mediated knockdown of four soluble factors in peripheral blood (PB)-derived NK cells. PB-NK cells were transfected with the control siRNA or the pooled siRNAs to reduce GPIbα, GM-CSF, TNFα and MIP-1α protein expression. Protein expression was measured after 48 hours of siRNA transfection. Knockdown of GPIbα protein expression was not assessed due to unavailability of quantitative assay. *P<0.01, **P<0.001, ns= not significant. Each dot represents one donor.

Supplementary Figure 6 Representative flow cytometry plot demonstrating cell purity before and after cell isolation. **(A)** Human PBMC were subjected to T and NK cell isolation as described in the Methods section and cells were stained with anti-CD3 (PE) and anti-CD56 (APC) to identify T and NK cells respectively. Before isolation, PBMC contained both T and NK cells, and following activation highly pure T and NK cells were obtained. CAR expression on the surface of NK cells **(B)** and T cells **(C)** was measured following incubating with Protein L.
