# Supplementary figures and images for: Soluble factors released by peripheral blood-derived CAR-NK cells cause bystander myeloid cell activation
Source: Front Immunol. 2024 Dec 24;15:1519415. doi: 10.3389/fimmu.2024.1519415 (PMC11703831; doi:10.3389/fimmu.2024.1519415)

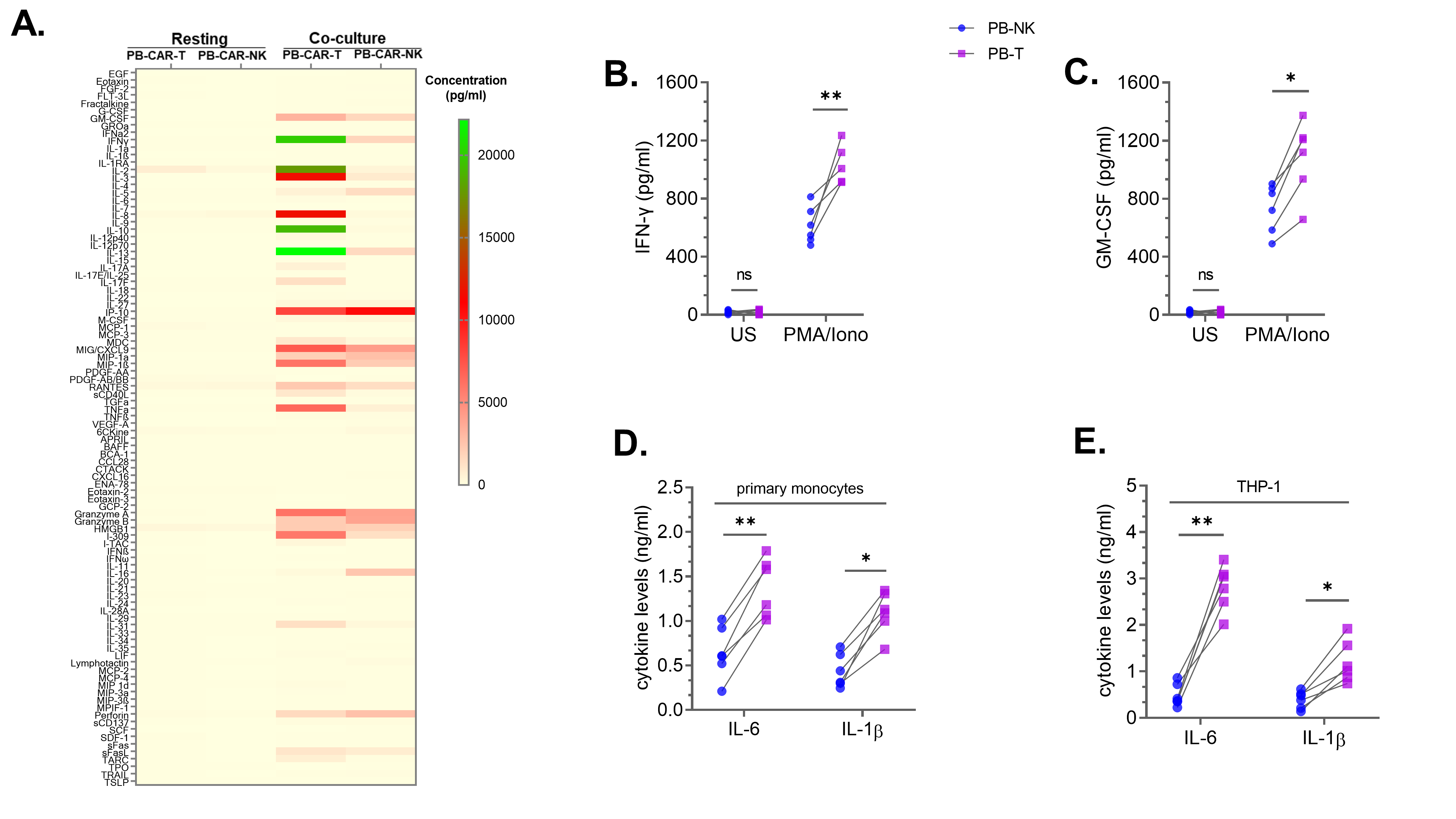

Supplement: Supplementary Figure 1 — Myeloid cell activation following CAR-independent activation of peripheral blood (PB)-derived NK (PB-NK) and T (PB-T) cells. (A) Heatmap of 96 inflammatory cytokines/chemokines present in the supernatant of resting or co-culture activated anti-CD19 PB-CAR-T cells and anti-CD19 PB-CAR-NK cells (E: T= 1:1). Cytokine/chemokine levels (expressed in pg/ml) are represented by a color gradient, ranging from yellow (no expression) to green (highest concentration). (B) IFNγ and (C) GM-CSF secreted by PB-NK cells and PB-T cells following CAR-independent activation using PMA/Ionomycin. Myeloid cell activation by activated PB-T and PB-NK cells was assessed by incubating supernatant from panel B with (D) donor-matched primary monocytes or (E) THP-1 cells and measuring IL-6 and IL-1 β released by myeloid cells. *P<0.05, **P<0.01. Each dot represents one donor, and the line represents cells from the same donor. [file Image1.tif]

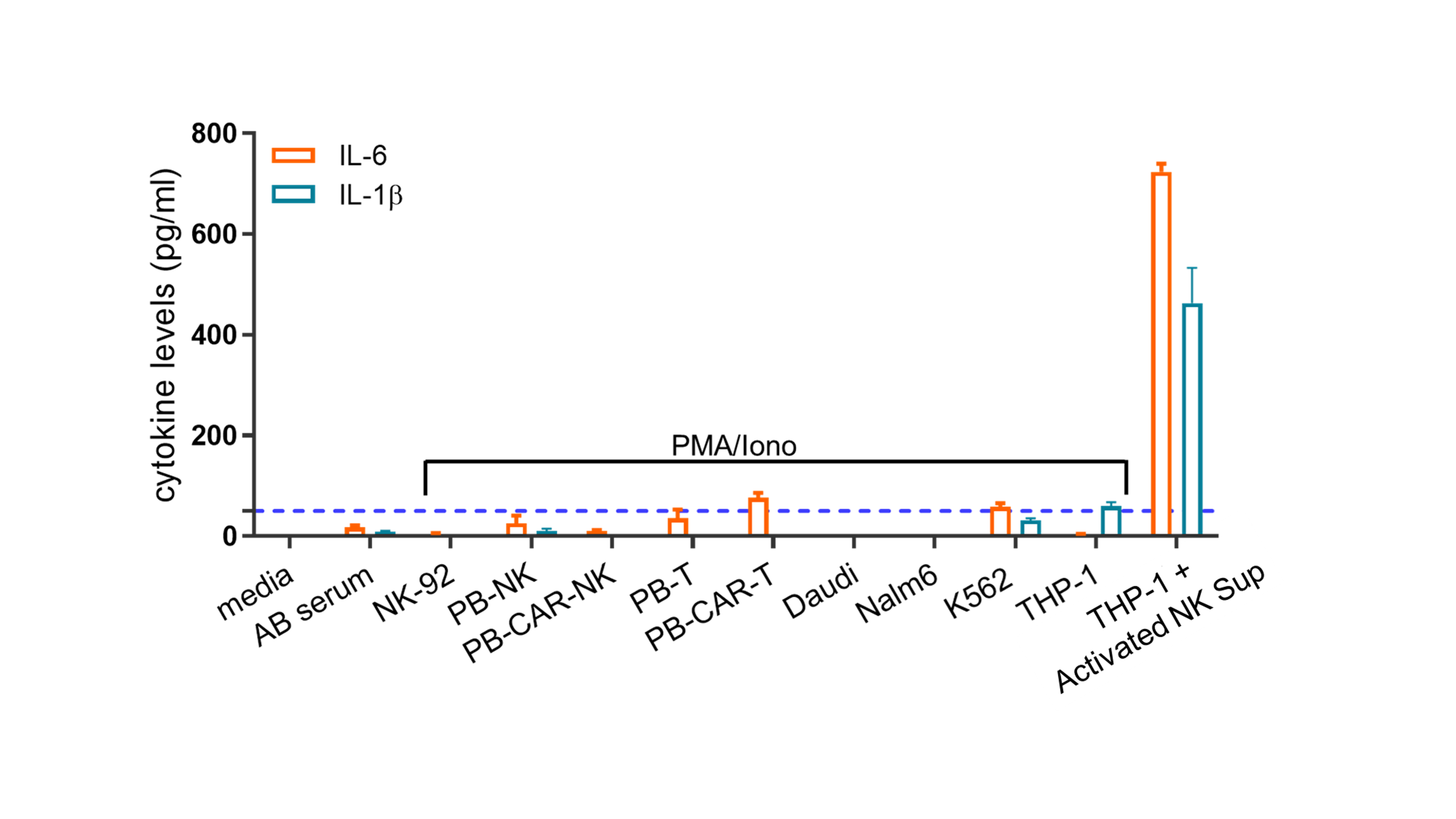

Supplement: Supplementary Figure 2 — Myeloid cells secrete IL-6 and IL-1β following stimulation with activated NK cell supernatant. IL-6 and IL-1β was measured in cell culture media, human AB serum used in cell culture and various cell types following PMA/Ionomycin stimulation or in THP-1 cells following stimulation with activated NK cell supernatant. None of the cells produced significant amount of IL-6 and IL-1β following PMA/Ionomycin stimulation suggesting these cells are not the major producer of these cytokines. Importantly, THP-1 cells also did not produce IL-6 and IL-1β following PMA/Ionomycin stimulation and IL-6 and IL-1β was only detected following stimulation of THP-1 cells with activated PB-NK cell supernatant. [file Image2.tif]

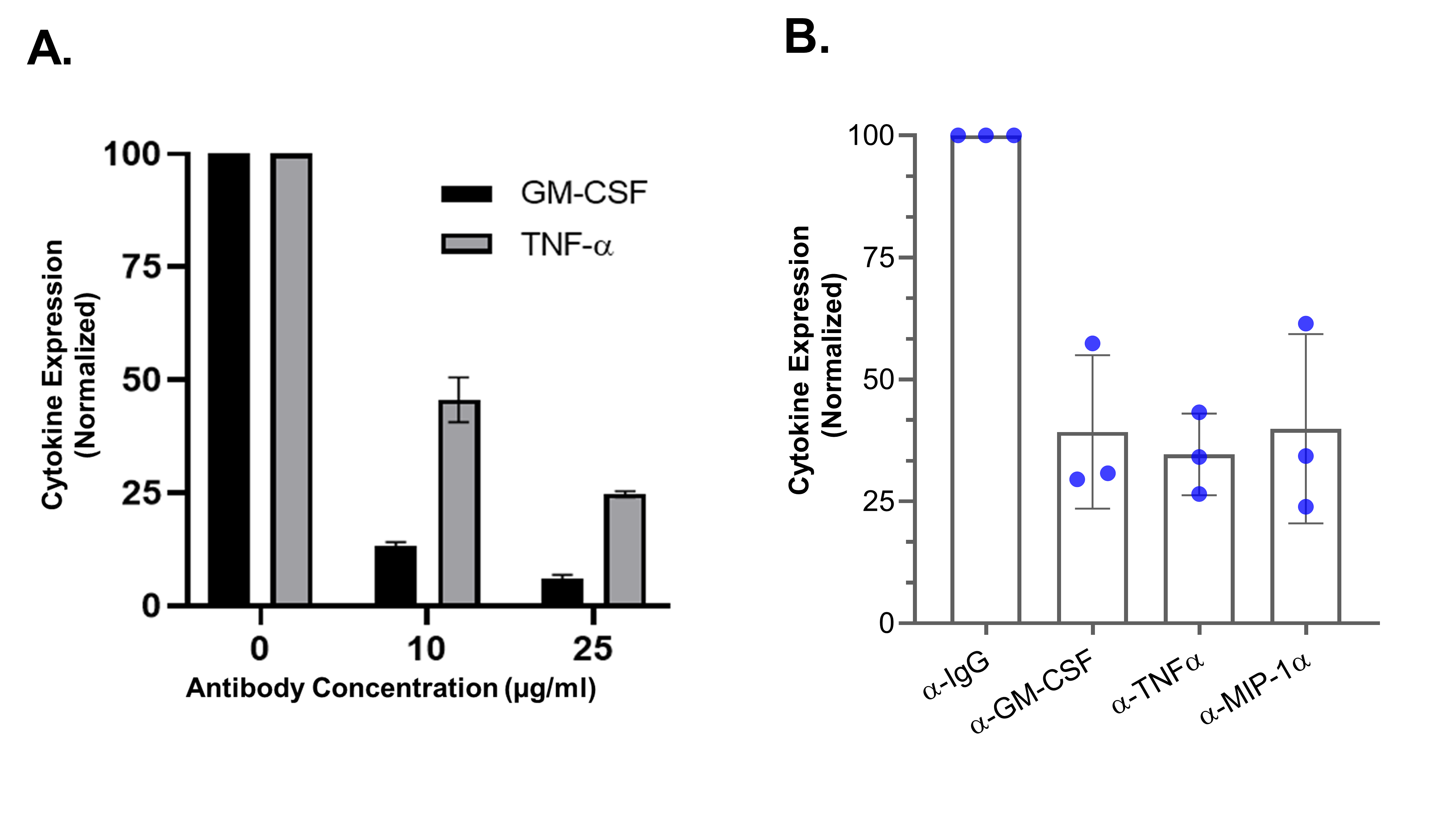

Supplement: Supplementary Figure 3 — Antibody-mediated neutralization of cytokines. (A) Activated PB-NK cell supernatants were incubated with either GM-CSF or TNFα antibodies at two different concentrations, 10 and 25 µg/ml. Efficiency of neutralization was assessed by measuring these cytokines in the neutralized supernatant by ELISA. (B) Activated PB-NK cell supernatant was incubated with pooled antibodies (GM-CSF, TNFα and MIP-1α) at 12.5µg/ml for each antibody. Efficiency of neutralization was assessed by measuring these cytokines in the neutralized supernatant by ELISA. Cytokine levels in the antibody neutralized supernatant was normalized to the levels present in the control IgG neutralized supernatant. Each dot represents one donor. [file Image3.tif]

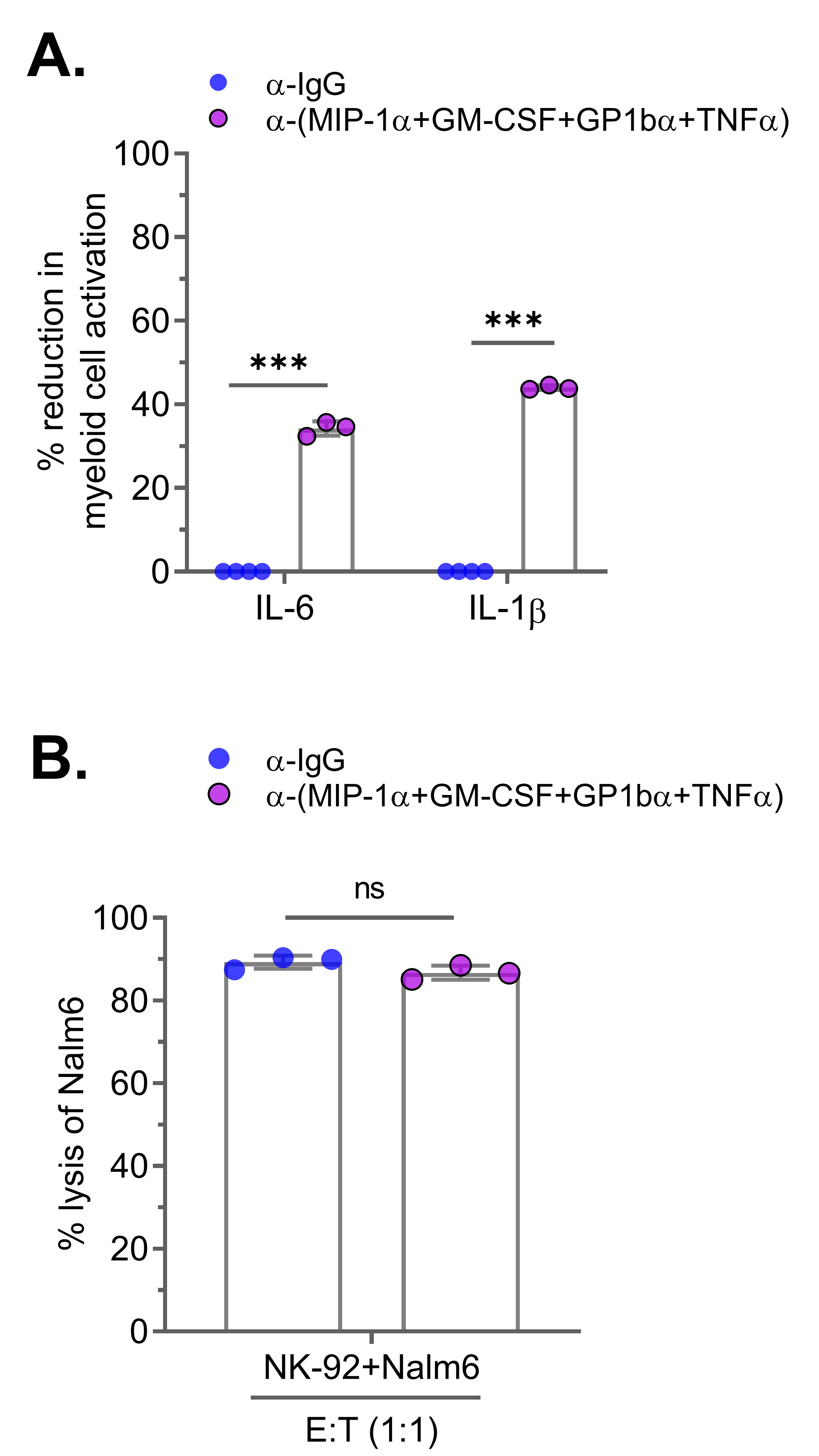

Supplement: Supplementary Figure 4 — Neutralization of four soluble factors in NK-92 cells reduces myeloid cell activation without affecting NK-92 cell potency. Supernatant obtained from Daudi co-culture (E: T= 1:1) activated NK-92 cells was subjected to antibody-mediated neutralization. Following neutralization, supernatant was added to the myeloid cells (THP-1) and secretion of IL-6 and IL-1β by myeloid cells were measured. Percent (%) reduction in myeloid cell activation was calculated by normalizing to the IL-6 and IL-1β secreted by myeloid cells incubated with IgG control treated NK-92 supernatant. (A) Percent (%) reduction in myeloid cell activation following neutralization of GPIbα, GM-CSF, TNFα, and MIP-1α in the supernatant obtained from activated NK-92 cells. (B) NK-92 cell potency was assessed by measuring lysis of Nalm6 target cells by NK-92 cells in the presence of control IgG or GPIbα, GM-CSF, TNFα, and MIP-1α antibodies. ***P<0.001, ns= not significant. Each dot represents one independent experiment. [file Image4.tif]

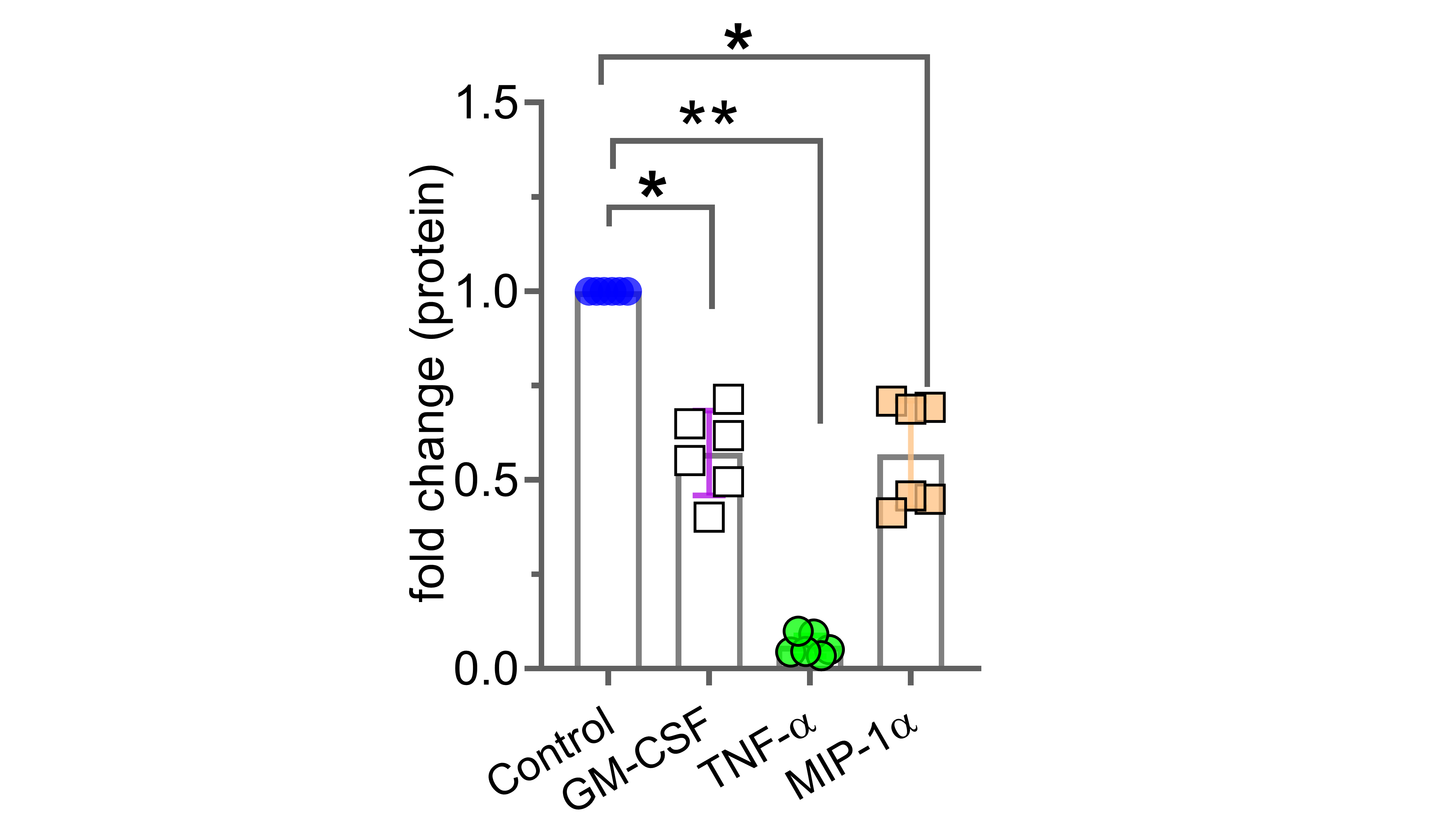

Supplement: Supplementary Figure 5 — Protein expression following siRNA-mediated knockdown of four soluble factors in peripheral blood (PB)-derived NK cells. PB-NK cells were transfected with the control siRNA or the pooled siRNAs to reduce GPIbα, GM-CSF, TNFα and MIP-1α protein expression. Protein expression was measured after 48 hours of siRNA transfection. Knockdown of GPIbα protein expression was not assessed due to unavailability of quantitative assay. *P<0.01, **P<0.001, ns= not significant. Each dot represents one donor. [file Image5.tif]

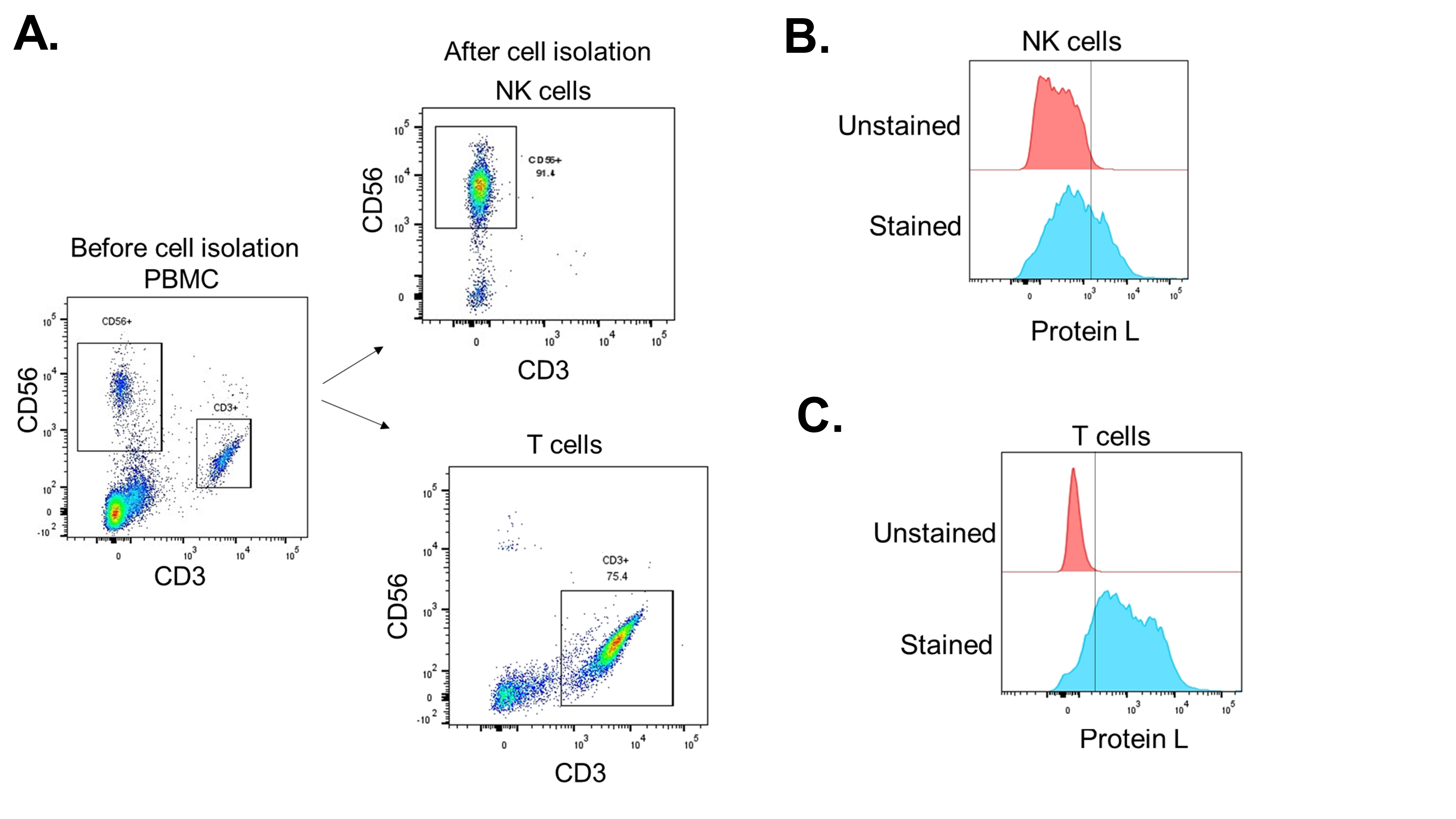

Supplement: Supplementary Figure 6 — Representative flow cytometry plot demonstrating cell purity before and after cell isolation. (A) Human PBMC were subjected to T and NK cell isolation as described in the Methods section and cells were stained with anti-CD3 (PE) and anti-CD56 (APC) to identify T and NK cells respectively. Before isolation, PBMC contained both T and NK cells, and following activation highly pure T and NK cells were obtained. CAR expression on the surface of NK cells (B) and T cells (C) was measured following incubating with Protein L. [file Image6.tif]
